# ATRX Alteration Contributes to Tumor Growth and Immune Escape in Pleomorphic Sarcomas

**DOI:** 10.3390/cancers13092151

**Published:** 2021-04-29

**Authors:** Lucie Darmusey, Gaëlle Pérot, Noémie Thébault, Sophie Le Guellec, Nelly Desplat, Laëtitia Gaston, Lucile Delespaul, Tom Lesluyes, Elodie Darbo, Anne Gomez-Brouchet, Elodie Richard, Jessica Baud, Laura Leroy, Jean-Michel Coindre, Jean-Yves Blay, Frédéric Chibon

**Affiliations:** 1 INSERM U1037, Cancer Research Center in Toulouse (CRCT), OncoSarc, 31000 Toulouse, France; lucie.darmusey@inserm.fr (L.D.); gaelle.perot@inserm.fr (G.P.); noemie.thebault@inserm.fr (N.T.); LeGuellec.Sophie@iuct-oncopole.fr (S.L.G.); lucile.delespaul@icr.ac.uk (L.D.); tom.lesluyes@crick.ac.uk (T.L.); brouchet.anne@chu-toulouse.fr (A.G.-B.); laura.leroy@inserm.fr (L.L.); 2 IUCT-Oncopole, Institut Claudius Régaud, Department of Pathology, 31000 Toulouse, France; 3 University of Toulouse 3, Paul Sabatier, 31000 Toulouse, France; 4 Centre Hospitalier Universitaire (CHU) de Toulouse, IUCT-Oncopole, 31000 Toulouse, France; 5 Inserm UMR1218, Action, Institut Bergonié, 33000 Bordeaux, France; N.Desplat@bordeaux.unicancer.fr (N.D.); elodie.darbo@u-bordeaux.fr (E.D.); E.Richard@bordeaux.unicancer.fr (E.R.); j.massiere@bordeaux.unicancer.fr (J.B.); j.coindre@bordeaux.unicancer.fr (J.-M.C.); 6 CHU de Bordeaux, Department of Medical Genetics, 33000 Bordeaux, France; laetitia.gaston@chu-bordeaux.fr; 7 University of Bordeaux, 33000 Bordeaux, France; 8 CNRS UMR5800, LaBRI, 33400 Talence, France; 9 Institut Bergonie, Department of Pathology, 33000 Bordeaux, France; 10 Centre Léon Bérard, Department of Medical Oncology, 69000 Lyon, France; Jean-yves.blay@lyon.unicancer.fr; 11 Inserm U1052, CNRS 5286, Cancer Research Center of Lyon, University Claude Bernard Lyon 1, 69000 Lyon, France

**Keywords:** sarcomas, ATRX, oncogenesis, mast cells, alteration

## Abstract

**Simple Summary:**

There is still no efficient systemic treatment for pleomorphic sarcomas. This study shows that 1/4 of them have an *ATRX* alteration that diminishes the immune response. This phenotype is related to the inhibition of mast cell recruitment upon *ATRX* alteration, which could be targeted to adapt immunotherapy against pleomorphic sarcomas.

**Abstract:**

Whole genome and transcriptome sequencing of a cohort of 67 leiomyosarcomas has been revealed *ATRX* to be one of the most frequently mutated genes in leiomyosarcomas after *TP53* and *RB1*. While its function is well described in the alternative lengthening of telomeres mechanism, we wondered whether its alteration could have complementary effects on sarcoma oncogenesis. *ATRX* alteration is associated with the down-expression of genes linked to differentiation in leiomyosarcomas, and to immunity in an additional cohort of 60 poorly differentiated pleomorphic sarcomas. In vitro and in vivo models showed that *ATRX* down-expression increases tumor growth rate and immune escape by decreasing the immunity load of active mast cells in sarcoma tumors. These data indicate that an alternative to unsuccessful targeting of the adaptive immune system in sarcoma could target the innate system. This might lead to a better outcome for sarcoma patients in terms of *ATRX* status.

## 1. Introduction

Pleomorphic sarcomas are a group of rare mesenchymal tumors comprising different histotypes, such as undifferentiated pleomorphic sarcoma (UPS), myxofibrosarcoma (MFS), dedifferentiated liposarcoma (DDLPS), osteosarcoma (OS), and leiomyosarcoma (LMS), which is the most frequent subtype [1]. LMS has a smooth muscle differentiation and can occur in any anatomical site, although there are three main locations: limbs, trunk, and uterus. Currently, the first-line treatment is wide-margin resection for localized tumors and anthracycline-based chemotherapy for advanced tumors [2]. However, these treatments are still not effective enough as 48 to 89% of LMS develop metastases depending on the tumor location, with a better outcome for patients with tumors in limbs, and the mortality rate is between 50 and 65% with a median survival of around 12 months [3,4]. From a genomic standpoint, LMS, like other pleomorphic sarcomas, have a very rearranged and unbalanced karyotype [2].

In a whole genome and whole transcriptome sequencing study conducted by our team, Darbo et al. showed that LMS could be separated into two groups with specific clinical, transcriptomic and genomic features: the homogenous and the other LMS. Those groups share a low somatic mutation burden and a high level of copy-number alterations [5]. But only three genes came out to be recurrently mutated (considering point mutations only), as also showed by the TCGA study [6]: *TP53*, *RB1*, and *ATRX* (mutated in 48.7%, 17.9%, and 12.8%, respectively). *RB1* [7,8] and *TP53* [9,10] are tumor suppressor genes that have long been known to be implicated in the oncogenesis of pleomorphic sarcomas. ATRX is a chromatin modifier gene with a Swi/Snf2 domain [11]. Its tumor suppressive function has so far been related to its role in the alternative lengthening of telomeres (ALT) mechanism, which is a way to elongate telomeres without telomerase activation [12], inducing genome instability [13] and leading to a poor prognosis of *ATRX*-altered tumors [14]. Recently, its involvement in senescence [15] and intrinsic immunity via its interactions within promyelocytic leukemia nuclear bodies (PML NBs) [16] was questioned. Here, we investigated whether ATRX might have additional impacts in the oncogenesis of pleomorphic sarcomas beyond its role in the ALT mechanism and show how its involvement in oncogenesis is also linked to differentiation, tumor growth, and immunity.

## 2. Material and Methods

### 2.1. Data Availability

Cohort 1: ICGC whole genome sequencing and RNA sequencing data for the 67 LMS are available at https://dcc.icgc.org/projects/LMS-FR.

Cohort 2: RNA-seq expressions are available on Gene Expression Omnibus under accession GSE71121. RNA-seq raw files (FastQ) are available on sequence read archive under accessions SRP059588 or SRP059588.

Mouse K7M2 tumor expression data are available on Gene Expression Omnibus (GEO) under accession GSE157953 and will be released on 09/01/2021 and available before by contacting the corresponding author.

### 2.2. Human Samples

Samples used in cohort 1 were collected prospectively by the French Sarcoma Group as part of the ICGC program (International Cancer Genome Consortium). Samples used in cohort 2 were part of the cohort used in Lesluyes et al. [17]. Clinico-pathological data and patient information are summarized in Table 1. All cases were systematically reviewed by expert pathologists of the French Sarcoma Group according to the World Health Organization guidelines [18]. In cohort 1, selected tumors were primarily untreated (before sampling), not superficial LMS without any additional criteria. In cohort 2, selected tumors were primarily untreated (before sampling) pleomorphic sarcoma. For each patient, frozen and FFPE samples of the primary tumor were collected before any treatment.

From those samples, DNA extraction, whole genome sequencing and analysis, RNA extraction, sequencing, and analysis, as well as annotation of variants and breakpoint detection can be found in the supplementary information and in Tables S4–S6.

### 2.3. Validation of ATRX Alterations

For cohort 1, all FS (frameshift) were verified at both DNA and RNA levels by Sanger sequencing. All SV (structural variants) were verified on DNA by Sanger sequencing, and the effect on RNA was detected by RNAseq. MS (missense) and NS (non-sense) mutations, not found in both Whole Genome sequencing and RNA sequencing, were verified by Sanger sequencing. Total loss of chrXq or chrX was seen in four females. This alteration induces a complete deletion of *ATRX*, but all cases expressed RNA expression and nuclear protein, implying that the loss occurred on the inactive X. One triploid tumor developed in a male also presented a deletion of the gene, but with one copy left. As one normal copy of the gene is expressed, they were all considered as wild type (WT) regarding *ATRX* alteration. For cohort 2, whole *ATRX* cDNA was sequenced by Sanger sequencing for all cases, and alterations found at RNA level were verified on DNA. Method details of PCR, RT-PCR, and Sanger sequencing can be found in the supplementary information.

Immunohistochemistry and immunofluorescence analyses are detailed in the supplementary information.

### 2.4. Cell Lines and Primary Culture

The cell lines MG63 (RRID:CVCL_0426; Male) and K7M2 (RRID:CVCL_V455; Female) were given by Dr. Françoise Redini. Those and HEK293T (RRID:CVCL_0063; Female) cells were cultured in DMEM (31-966-021, Life Technologies, Carlsbad, CA, USA). IB106 (UPS; Female) cell is a primary culture established as previously described [19] and was cultured in RPMI-1640 (524-000-025, Life Technologies, Carlsbad, CA, USA). Both medium were supplied with 10% fetal bovine serum (S1810-500, Dutscher, Brumath, France), and cells were kept at 37 °C in a humidified chamber containing 5% CO_2_.

Production and validation of stable ATRX knock-down cell lines, as well as in vitro analyses, can be found in the supplementary information.

### 2.5. In Vivo Experiment

All experiments were performed in conformity with the rules of the French Institutional Animal Care and Use Committee (approval number DAP-APAFiS-2018041617309605), and all efforts were made to minimize animal suffering. Mice were maintained under specific pathogen-free conditions in the animal facility of the University of Bordeaux (Bordeaux, France) or at the CREFRE (Centre Régional d’Exploration Fonctionnelle et Ressources Expérimentales, Toulouse, France).

For experiments with IB106 cells, 6–8-week-old female NSG (NOD.Cg-Prkdc^scid^ Il2rg^tm1Wjl^/SzJ; RRID:BCBC_4142) mice were used. Ten mice were injected with 800,000 IB106 *ATRX^KD^* cells, and ten with 800,000 IB106 *ATRX^CT^* cells as controls. Mice were randomly assigned to one cage of five animals then each cage was randomly assigned to a group of cells. One mouse in the control group was excluded, due to an important and quick loss of weight, so the final number of units is *n* = 10 in the *ATRX^KD^* group and *n* = 9 in the *ATRX^CT^* one. Tumor sizes were blindly measured twice a week using a caliper, and their volume was calculated using the formula: (L^2^ × l)/2. At the end of the experiment, mice were sacrificed by cervical dislocation. Tumors were then weighed and divided into two parts for formalin fixation and nitrogen freezing. Each tumor was stained with HE and analyzed by a pathologist specialized in sarcomas. Growth rates were calculated with the segmental linear regression of GraphPad Prism (GraphPad Software, version 6, San Diego, CA, USA), and statistical analyses were done using an unpaired T-test.

For experiments with K7M2 cells, 6–8 weeks-old female NSG (NOD.Cg-Prkdc^scid^ Il2rg^tm1Wjl^/SzJ; RRID:BCBC_4142) or Balb/c (Balb/cJ; RRID:IMSR_JAX:000651) mice were used. Four groups were made, each group was composed of 15 mice, one group of NSG mice was injected with 500,000 K7M2 *ATRX^KD^* cells, the other with 500,000 K7M2 *ATRX^CT^* cells as controls. The Balb/c mice were injected in the same conditions. Mice were randomly assigned to one cage of five animals then each cage was randomly assigned to a group of cells. One animal from the Balb/c *ATRX^CT^* group was excluded, due to a teeth malformation and an incapacity to eat, so the final number of units was *n* = 15 in each group except in this one which was *n* = 14. Tumor sizes were measured without knowing the affiliated group twice a week using a caliper, and their volume was calculated using the formula: (L^2^ × l)/2. At the end of the experiment, mice were sacrificed by cervical dislocation. Tumors were then weighed and divided into two parts for formalin fixation and nitrogen freezing. Each tumor was stained with HE and analyzed by a pathologist specialized in sarcomas. Growth rates were calculated with the segmental linear regression of GraphPad Prism (GraphPad Software, version 6, San Diego, CA, USA), and statistical analyses were done using an unpaired T-test. Survival curves were analyzed with GraphPad Prism using the Kaplan-Meier method.

### 2.6. Mice Tumor RNA Sequencing and Analysis

Total RNA was extracted, prepared, and sequenced as described in the supplementary information to obtain more than 20 million paired-end reads with a length of 75 bp each. Bioinformatic analysis was done as previously described [17].

RNA reads were aligned to the mm10 genome assembly with STAR v2.6.0c [20]. Low-quality (score < 20) and duplicated PCR paired-end reads were removed with SAMtools v1.8 [21] and PicardTools v2.18.2 [22] (http://broadinstitute.github.io/picard/), respectively. Then, gene expression was quantified with Cufflinks v2.2.1 [23], using RefSeq [24] genes (without miRNA and rRNA) from mm10 UCSC Table Browser [25] fixed on 2019/01.

Differential gene expression was performed by R package DESeq, between *ATRX^KD^* and *ATRX^CT^* tumors extracted from Balb/c mice. Relationships between proteins overexpressed in *ATRX^KD^* and *ATRX^CT^* tumors were assessed by the STRING Database [26].

### 2.7. Quantification and Statistical Analysis

Kaplan-Meier analyses were performed for metastasis-free survival and overall survival. To subdivide *ATRX* expression into two groups, expression was plotted for *ATRX* WT and altered cases, separately. The intersection between these two density curves was 4.45 (log2 FPKM) and 2.77 for cohort 1 and 2, respectively.

Differential gene expression (DGE) analyses were performed by R package DESeq. Gene Ontology (GO) analysis was performed on these differentially expressed genes (*p* < 0.05 and fold-change >2 or <−2), by R package GOseq. In parallel, significant genes with *p* < 0.01 were used to make a heatmap (R package ComplexHeatMap).

Every other statistical analysis detail can be found in each figure legend.

## 3. Results

### 3.1. Distinct Genetic Alterations Trigger Loss of ATRX Protein in Leiomyosarcomas

**Figure 1:**
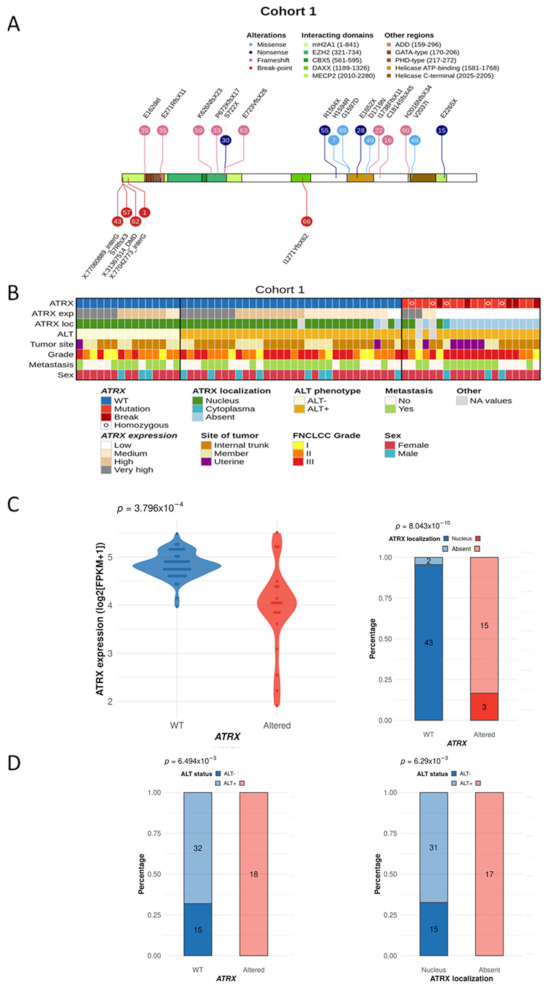
ATRX status and integrated representation in leiomyosarcomas. (**A**) *ATRX* alterations are color-coded according to their type (legend at the top). Numbers in bubbles represent tumor samples. Consequences of all point mutations on ATRX protein are annotated above a schematic representation of the protein, or below for two structural variants (LMS57 and LMS66). For the other three structural variants, annotations correspond to the break-point partner in genomic coordinates. (**B**) The integrated representation shows *ATRX* alterations, *ATRX* mRNA expression (by quartile), ATRX localization, ALT mechanism phenotype, tumor site, FNCLCC grade, presence or not of metastasis, and sex of each patient. Tumors are ordered by *ATRX* status, ALT phenotype, mRNA expression, and protein localization. (**C**) Association between *ATRX* alteration and its mRNA expression (log2 (FPKM + 1)) (left) or its protein localization (right). (**D**) Relation between *ATRX* status (left) or its protein localization (right) and ALT mechanism phenotype. For ATRX localization, the “absent” group means “not at the nucleus”, including all cases without expression and the case with a cytoplasmic localization (LMS16). *p*-values were calculated with Student test for (**C**)—left and with Fisher test for (**C**)—right and (**D**).

Sixty-seven LMS (Cohort 1; Table 1) were sequenced at the whole genome, and transcriptome levels (67 LMS) and *ATRX* was identified as the third most frequently mutated gene after *TP53* and *RB1*. By integrating point mutations and SV, ATRX is altered in 20 cases (29.8%; Figure 1A), with 8 point mutations (MS and NS; 40%), 7 FS (35%) and 5 SV (25%). All mutations and SV were validated by an independent technique (RNA sequencing and/or Sanger sequencing) (Tables S1 and S2). *ATRX* was altered in 23.7% of non-uterine LMS (14/59) compared to 75% of uterine LMS (6/8), which is significantly higher in this specific anatomical site (*p* = 0.007; Figure 1B and Figure S1). Altered cases were not enriched in any other clinical annotation (i.e., grade, metastasis, or sex). Regarding SV, 3 out of 5 led to a loss of *ATRX* expression, and the other two led to an FS (Table S1). These alterations were hemizygous in the three males, due to the location of *ATRX* on chromosome X (Xq21), and in two females with either deletion of the second allele (LMS69) or an isodisomy (LMS49). In the other 15 females, 93.3% of the alterations (14/15) occurred on the active X, as RNAseq analysis showed the altered transcript expression (Table S1). No expression of the mutated allele was detected in LMS48 (Figure 1B and Table S1). Alterations were distributed throughout the whole gene, but two regions were most frequently affected: one between exons 17 and 21 (40%, 6/15) and the other in exon 9 (33.4%, 5/15). At the mRNA level, mutated cases had a significantly lower *ATRX* expression than WT tumors (*p* = 0.000379; Figure 1C), and at the protein level, alterations led to a loss of nuclear protein (*p* < 0.0001; Figure 1C and Figure S2).

### 3.2. ATRX Alteration Is Linked to ALT Mechanism in Leiomyosarcomas

Since *ATRX* loss is linked to the ALT phenotype [27], the ALT status of tumors was determined. Most LMS were ALT-positive (ALT+, 76.9%, 50/65) (Figure 1B and Figure S3). Both *ATRX* alteration (*p* = 0.00649) and ATRX protein loss (*p* = 0.00629) were significantly associated with the ALT mechanism (Figure 1D). However, while all *ATRX*-altered cases were ALT+, most ALT+ cases were *ATRX* WT (64%, 32/50), with 93.3% of cases (28/30) expressing the protein in the nucleus (Figure 1B,D).

### 3.3. ATRX Alteration Is Not Associated with Prognosis in Leiomyosarcomas

Neither *ATRX* status (altered or WT), mRNA expression (below or above defined cut-off, see material and methods section), protein localization (nuclear or absent), nor ALT phenotype (positive or negative) could split patients into two groups with significantly distinct prognoses (Figure S4).

### 3.4. Differentiation Transcriptomic Programs Is Modified upon ATRX Alteration in Leiomyosarcomas

**Figure 2:**
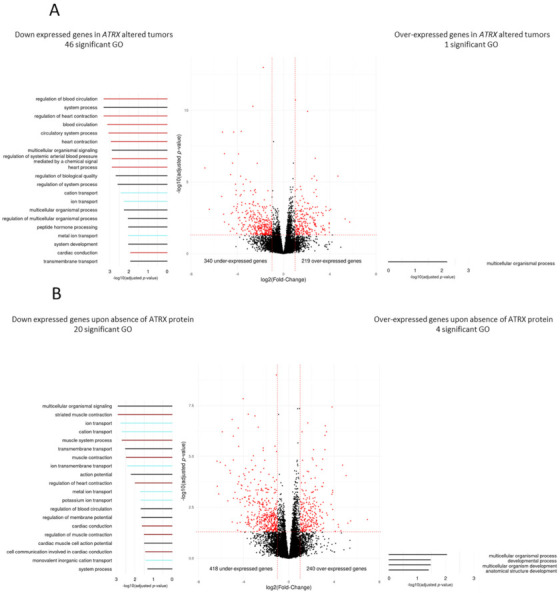
Differential gene expression and Gene Ontology analyses according to ATRX alteration in leiomyosarcomas (Cohort 1). Differentially expressed genes according to (**A**) *ATRX* status (wild-type vs. altered) or (**B**) ATRX expression (nucleus vs. absent). Red dots indicate significant genes (*p* ≤ 0.05 and fold-change ≤−2 or ≥2). Gene Ontology (GO) analyses, represented on the left (under-expressed genes) and the right (over-expressed genes), identified 46 and 1 significant GO terms (*p* ≤ 0.05), respectively in (**A**) and 20 and 4 significant GO terms (*p* ≤ 0.05) in (**B**). On each side, the 20 most significant GO terms are represented and color-coded by mechanisms; light red, dark red, light blue, and black colors indicate “circulatory system process”, “muscle system process”, “ion transport” and general terms, respectively. For ATRX localization, the “absent” group means “not at the nucleus”, including all cases without expression and the case with a cytoplasmic localization (LMS16). All *p*-values were adjusted by Benjamini and Hochberg method.

Searching for the oncogenic impact of these *ATRX* alterations, we tested whether altered tumors had a distinct transcriptomic program and identified 340 and 219 genes significantly down- and up-expressed in the *ATRX*-altered group, respectively (*p* < 0.05; Figure 2A). Functional enrichment analysis (Figure 2A) showed that genes down-expressed were significantly involved in blood pressure, heart contraction, and striated muscle contraction. These findings were strengthened when patients were grouped according to ATRX protein localization, since genes down-expressed upon protein loss were found to be involved in similar biological mechanisms, i.e., muscle system and contraction (Figure 2B).

As expected, clustering based on these 559 differentially expressed genes (Figure S5A) revealed a group with a high percentage of *ATRX*-altered patients (75%, 15/20). Patients in this cluster had tumors that were enriched in uterine or “other” LMS type (*p* < 0.0001; Figure S5B) [5]. “Other” LMS are less differentiated than “homogeneous” LMS and are thought to derive from fibroblasts rather than smooth muscle cells (SMC) [5].

The association between enrichment of down-expressed genes linked to muscle system and of oLMS in ATRX altered tumors suggested that either *ATRX* alteration preferentially occurs in partially or undifferentiated cells, or that it may induce dedifferentiation. To explore these hypotheses, we studied the *ATRX* status in a second cohort comprising poorly differentiated pleomorphic sarcomas characterized by RNAseq.

### 3.5. ATRX Alterations Are Recurrent and Similar in Poorly Differentiated Pleomorphic Sarcomas

**Figure 3:**
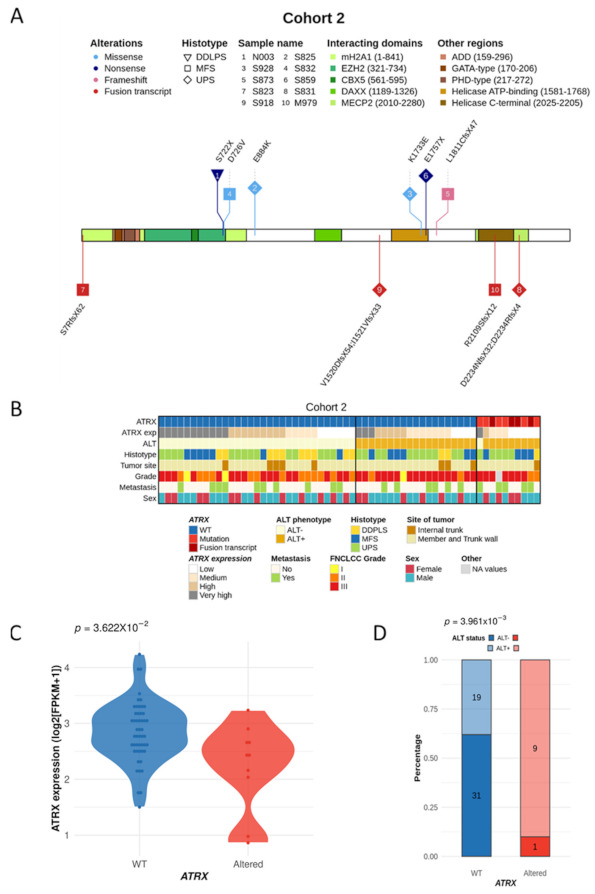
*ATRX* alterations and integrated representation in poorly differentiated pleomorphic sarcomas (cohort 2). (**A**) *ATRX* alterations are color-coded by their type, and shapes represent histotypes. Numbers in bubbles indicate a tumor sample (legend at the top). Translated consequences on ATRX protein is annotated above a protein schematic representation for mutations, or be-low for fusion transcripts. (**B**) Integrated representation shows *ATRX* alterations, mRNA expression (by quartile), ALT mechanism phenotype, histotypes, tumor site, FNCLCC grade, presence or not of metastasis and sex of each patient. Tumors are ordered by *ATRX* status, ALT phenotype, mRNA expression and histotypes. (**C**) Association between *ATRX* status and its mRNA expression (log2(FPKM+1)). (**D**) Relation between *ATRX* status and ALT phenotype.

RNA sequencing of 60 poorly differentiated pleomorphic sarcomas (cohort 2; Table 1) from a previously published cohort [17] was reanalyzed, and 10 *ATRX*-altered tumors (16.7%) were identified (Tables S1 and S3). The types of alteration, as well as their functional consequences, were similar to those detected in cohort 1 (Figure 3A). Altered cases were not enriched in any annotation (i.e., histotype, tumor site, grade, metastasis, or sex) (Figure 3B), but had a significantly lower mRNA expression of *ATRX* (*p* = 0.0362; Figure 3C) and were significantly associated with ALT (*p* = 0.00396; Figure 3D). *ATRX*-altered tumors did not have a distinct prognosis in cohort 2 (Figure S6A), nor when the two cohorts were merged (Figure S6B). 

### 3.6. Immunity Transcriptomic Program Is Modified upon ATRX Alteration in Poorly Differentiated Pleomorphic Sarcomas

**Figure 4:**
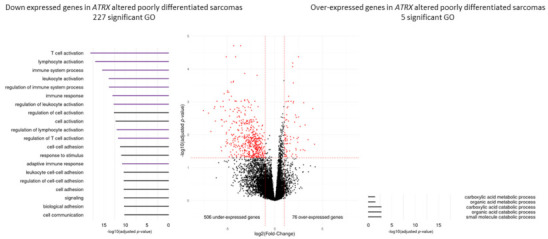
Differential gene expression and Gene Ontology analyses according to *ATRX* status (wild-type vs. altered) in poorly differentiated pleomorphic sarcomas (Cohort 2). Differentially expressed genes in *ATRX*-altered tumors are represented in red (*p* ≤ 0.05 and fold-change ≤−2 or ≥2). Gene Ontology (GO) analyses, represented on the left (under-expressed genes) and the right (over-expressed genes), identified 227 and five significant GO terms (*p* ≤ 0.05), respectively. On the left, the 20 most significant GO terms are represented and color-coded by mechanism; purple and black groups indicate “immunity system process” and general terms, respectively. All *p*-values were adjusted by Benjamini and Hochberg method.

Functional enrichment analysis of differentially expressed genes showed that *ATRX* alteration induced the overexpression of 76 genes enriched in GO terms related to the metabolic process, and the down-expression of 506 genes enriched in GO related to immunity. The five most significantly enriched GO were (Figure 4) “T cell activation” (*p* < 0.0001), “lymphocyte activation” (*p* < 0.0001), “immune system process” (*p* < 0.0001), “leukocyte activation” (*p* < 0.0001) and “regulation of immune system process” (*p* < 0.0001).

Results from both cohorts indicated that *ATRX* alteration was associated with differentiation and immunity. Since this is particularly relevant as immunotherapies are currently not efficient in pleomorphic sarcomas, we functionally tested the hypothesis that *ATRX* alteration might modify the anti-tumor immune response. 

### 3.7. ATRX Knock-Down Impact Oncogenic Features toward Aggressiveness

**Figure 5:**
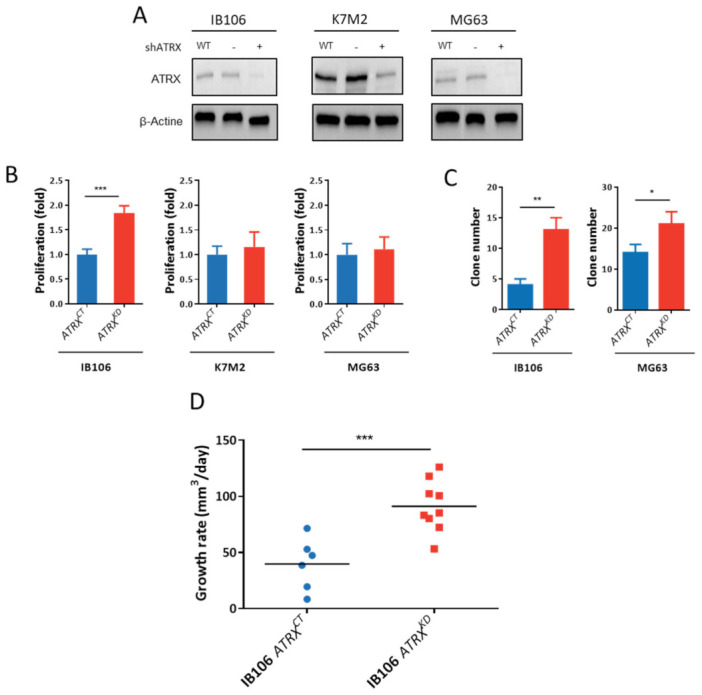
*ATRX* knock-down increases the aggressiveness of sarcoma cells. (**A**) *ATRX* knock-down by shRNA validation in western blot in K7M2, MG63, and IB106 cell lines. (**B**) Proliferation analysis by MTT after four days, comparing *ATRX^CT^* and *ATRX^KD^* cells in K7M2, MG63, and IB106 cell lines (mean ± s.d.; *n* = 3 independent experiments). (**C**) Soft agar assay analysis comparing *ATRX^CT^* and *ATRX^KD^* cells in K7M2, MG63, and IB106 cell lines (mean ± s.d.; *n* = four independent experiments). Images were taken after four weeks and crystal violet staining. (**D**) Tumor growth rate analysis of IB106 *ATRX^CT^* or IB106 *ATRX^KD^* cells sub-cutaneous xenografts on NSG mice (*n* = 10 in each group). The growth rate was calculated by segmental linear regression with GraphPad. * *p* ≤ 0.05, ** *p* ≤ 0.01, *** *p* ≤ 0.001, *p*-value was calculated with 2-way ANOVA for (**A**) and unpaired *t*-test for (**B**–**D**).

To functionally test the impact of *ATRX* alterations, three models of *ATRX* knock-down (*ATRX^KD^*) were constructed: (i) A model to evaluate tumor growth in vitro and in vivo in a human UPS cell line (IB106), (ii) another to study immune response a syngeneic model was necessary, given that no such model exists for LMS or UPS, we used the mouse poorly differentiated osteosarcoma K7M2 (only available Balb/c syngeneic mouse cell line of poorly differentiated sarcoma with very close genetics to UPS and LMS) and (iii) a third to compare mouse osteosarcoma (K7M2) behavior to human, using a human osteosarcoma cell line (MG63). These cell lines were transduced by lentivirus with an *ATRX* shRNA. Western blot evidenced the successful extinction of ATRX in each cell line (Figure 5A). ALT analysis showed that *ATRX* shRNA did not change ALT status in any cell line (Figure S7A).

In vitro, a significant (*p* < 0.0001) increase in proliferation was observed in the UPS cell line IB106 *ATRX^KD^*, but not in OS cell lines (K7M2 and MG63) (Figure 5B). Colonies formed in soft agar assay revealed that the mouse cell line K7M2 was unable to form any colony with or without ATRX expression. In contrast, there was a significant increase in colony number in human cell lines IB106 and MG63 upon *ATRX* down-expression, from a mean of 14 to 21 colonies (*p* = 0.0026) and from 4 to 13 (*p* = 0.001), respectively (Figure 5C). Next, IB106 *ATRX^CT^* (control) and *ATRX^KD^* cells were subcutaneously grafted in 10 NSG mice each. A tumor grew in the 6/9 *ATRX^CT^* group and the 9/10 *ATRX^KD^* group. Tumor growth rates were three-fold higher in *ATRX^KD^* tumors (91.2 ± 7.6 mm^3^/day) than in *ATRX^CT^* tumors (32.9 ± 10.6 mm^3^/day) (*p* = 0.0005; Figure 5D).

### 3.8. ATRX Knock down Modifies Anti-Tumor Immune Response In Vivo

**Figure 6:**
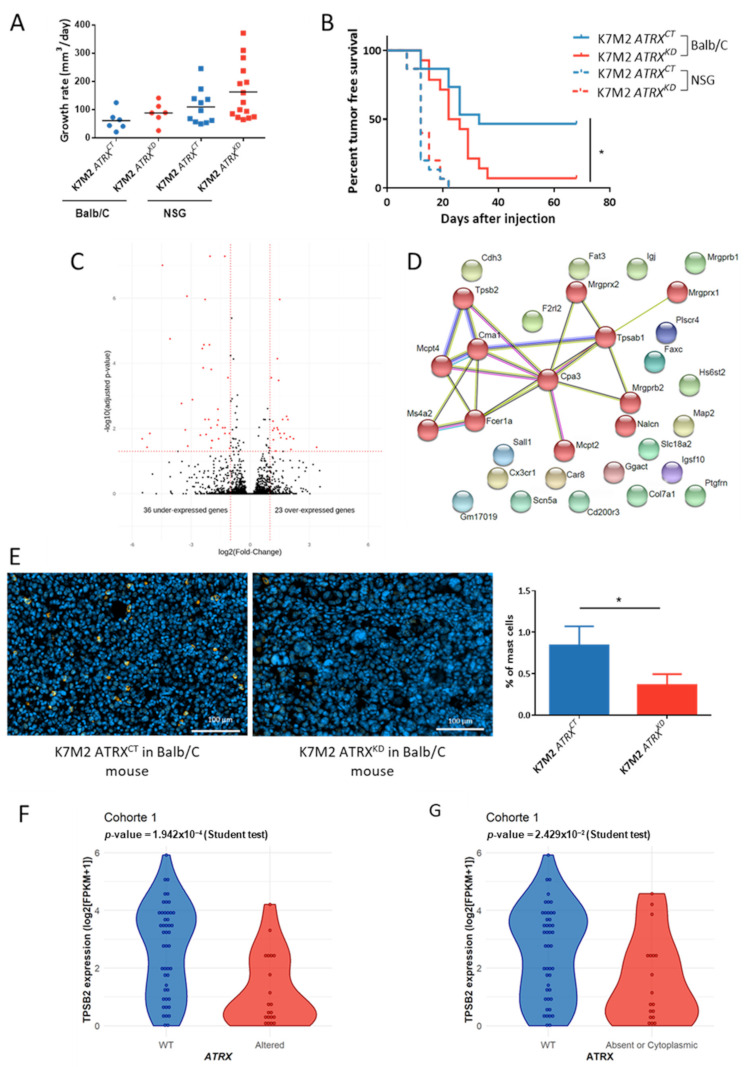
*ATRX* knock-down allows immune escape of sarcomas via non-recruitment of mast cells. (**A**) Tumor growth rate analysis of K7M2 *ATRX^CT^* or K7M2 *ATRX^KD^* cells xenografted under the skin of NSG or Balb/c mice (*n* = 15 in each group). (**B**) Tumor-free survival curves of K7M2 *ATRX^CT^* or *ATRX^KD^* tumors in immunodeficient NSG mice and immunocompetent Balb/c mice (*n* = 15 mice for each condition) using Kaplan-Meier method. (**C**) Comparison of RNA expression in log2 (FPKM + 1) of K7M2 *ATRX^KD^* tumors versus K7M2 *ATRX^CT^* tumors developed in immunocompetent mice (*n* = 4 each) showing 23 and 37 significantly up- and down-expressed genes in K7M2 *ATRX^KD^* tumors, respectively. (**D**) Links between down-expressed genes in K7M2 *ATRX^KD^* tumors found by the STRING Database showing one cluster with genes involved in mast cells via MCL clustering. (**E**) Immunostaining of mast cells by targeting tryptase in K7M2 *ATRX^CT^* and K7M2 *ATRX^KD^* tumor tissues with nucleus marked with DAPI. On the right, percent of mast cells in the two conditions. (**F**) *TPSB2* mRNA expression in log2 (FPKM + 1) according to *ATRX* status in cohort 1. (**G**) *TPSB2* mRNA expression in log2 (FPKM + 1) according to ATRX localization in cohort 1. * *p* ≤ 0.05, *p*-value was calculated with Mantel-Cox test for (**B**) and unpaired *t*-test for (**E**–**G**).

The involvement of ATRX alteration in the immune escape was tested by grafting K7M2 *ATRX^CT^* and *ATRX^KD^* cells in immunodeficient NSG mice and immunocompetent Balb/c mice (*n* = 15 for each group). The growth rate was not significantly increased upon ATRX knock-down in any hosts (Figure 6A). Tumor-free survival in *ATRX^CT^* and *ATRX^KD^* models displayed no significant differences in immunodeficient NSG mice, whereas in immunocompetent Balb/c mice, there was 53.4% (8/13) of tumor induction with K7M2 *ATRX^CT^ versus* 92.8% (13/14) with K7M2 *ATRX^KD^*. Therefore, tumor-free survival was significantly poorer upon *ATRX* knock-down (*p* = 0.0097; Figure 6B). These results display a higher likelihood of developing a poorly differentiated sarcoma with a low *ATRX* expression in an immunocompetent host.

Differential gene expression analysis between *ATRX^KD^* and *ATRX^CT^* K7M2 tumors in Balb/c mice revealed that 37 genes were down-regulated and 23 genes were overexpressed in *ATRX^KD^* tumors (Figure 6C). The low number of genes precluded any functional enrichment analysis. Consequently, a String Protein Interaction [26] analysis was performed. Whereas, no consistent clusters arose with up-regulated genes upon *ATRX* knock-down (Figure S7B), one emerged in down-regulated genes, with 12 proteins out of 37 linked to mast cell pathways (including *TPSB2* coding tryptase, a widely used mast cells marker) (Figure 6D).

Immunofluorescence against tryptase on the murine tumors previously processed in RNAseq showed that mast cells expressing tryptase represented a mean of 0.8% of total cells in *ATRX*^CT^ tumors, whereas they constituted 0.3% of *ATRX*^KD^ tumors (*p* = 0.01; Figure 6E). This significant difference prompted us to assess whether the proportion of infiltrating mast cells in human sarcomas is also related to *ATRX* alteration and the absence of ATRX from the nucleus. In the undifferentiated sarcoma cohort, *TPSB2* expression is decreased when ATRX is altered (based on RNAseq analysis) without reaching significance (*p* = 0.32; Figure S7C). Whereas, in the LMS cohort, which is deeply and fully characterized at both genome and protein levels for ATRX alteration, *TPSB2* was significantly under-expressed in *ATRX*-altered cases (*p* = 0.00019; Figure 6F) and in tumors with no nuclear ATRX (*p* = 0.02; Figure 6G). These results indicate that there are most probably fewer infiltrating mast cells in these human sarcoma subtypes.

## 4. Discussion

This in-depth *ATRX* genetic analysis revealed that *ATRX* alteration likely affects a quarter of pleomorphic sarcomas, since it was found in 29.8% of LMS and 16.7% of poorly differentiated pleomorphic sarcomas. Cohort 2 is less deeply characterized (WGseq for cohort 1, RNAseq for cohort 2), so cases might be missed with this RNAseq-based screening (20.7% observed for UPS/MFS/DDLPS in TCGA [6]). The rate of alteration in LMS is consistent with the rate of 24% found by Chudasama and colleagues [28], but it is slightly higher than that generally observed in other LMS cohorts, which is around 16% [6,29,30], probably due to the exhaustiveness of WGseq. *ATRX* mutations were distributed across the entire gene, as previously observed [6,28,31,32]. Three main factors link the two types of sarcomas in the present study: (i) In females, all alterations except in LMS48 can be interpreted as occurring on the active X, (ii) point mutations are more frequent (75% in LMS and 60% in the US) than structural variations (25% in LMS and 40% in the US), as previously observed [6,28]; and (iii) the alterations lead preferentially to a frameshift, and thus, to a truncated protein in 66.7% of cases (20/30, 65% in LMS and 70% in the US), in agreement with previous descriptions in sarcomas [6,28,31,33]. Of note, *ATRX* alterations in the present study were not significantly associated with a poorer prognosis. However, this association depends on the cohorts studied [14,32] and was observed in only one cohort that mainly included missense mutations [32].

*ATRX* mutated cases were also linked to the location of LMS, i.e., 75% of uterine cases were *ATRX*-altered (6/8) which is consistent with the rate found by Slatter and colleagues in uterine LMS. In this cohort of uterine LMS (26 uterine LMS), *ATRX* loss was linked to a poorer prognosis [34]. In our cohort, all altered *ATRX* uterine LMS metastasized and 1 out of 2 in the *ATRX* wild-type group, but the limited number of uterine LMS does not allow us to do any statistical analysis. Loss of *ATRX* in uterine tumors is a key difference between benign and malignant tumors. In this location, it has been proposed to use *ATRX* loss as a marker of the highly probable evolution of benign tumors toward malignancy [35]. In other LMS locations, ATRX loss is linked to the “other” LMS group. LMS belonging to this subtype are mainly poorly differentiated and likely originate from fibroblastic cells [5]. Furthermore, as ATRX loss in LMS is associated with a lower expression of genes related to smooth muscle activity, we hypothesize that it occurs preferentially in poorly differentiated cells. The degree of cell differentiation may be crucial for the loss of ATRX to confer advantages to the precursor leiomyosarcoma cell.

*ATRX* knock-down modifies tumor cell proliferation, as confirmed in vivo, where *ATRX* knock-down tumors grew three-fold faster than controls, and clonogenicity in sarcoma models. Interestingly, poorly differentiated pleomorphic sarcomas with *ATRX* alteration overexpressed genes related to metabolism, whose upregulation is a known hallmark of cancers and supports cell survival and proliferation [36]. The hypothesis that ATRX could act through metabolism regulation is a very appealing one that now requires functional validation.

In vivo experimentation revealed a new role of ATRX, as its alteration was associated with a poorer outcome exclusively in an immunocompetent murine host, and with down-expression of immune-related genes in poorly differentiated pleomorphic human sarcomas. These two findings show that ATRX loss can influence the regulation of immune response in sarcomas, probably by limiting mast cell recruitment, as evidenced by the lower proportion of tumor-infiltrating mast cells upon *ATRX* down-expression. The role of mast cells in tumor control is currently considered as dual and antagonistic since they can support tumorigenesis or suppress tumor growth. Their role is dependent on the type of tumor [37]. To our knowledge, no study has yet investigated the role of mast cells in the oncogenesis of sarcomas. *FcεRI* and *Ms4a2* are two down-expressed genes in *ATRX^KD^* K7M2 tumors. They are part of the IgE activating mast cell pathway that confers them a protective role in epithelial tumors [38]. In addition to their higher proportion, these mast cells present in *ATRX^WT^* tumors likely play a suppressor role in recruiting other immune cells to tumor sites by enhancing vascular permeability and direct chemoattraction [39]. In human LMS, the absence of ATRX is linked to the down-expression of *TPSB2*, which is a protein produced almost exclusively by mast cells and widely used to identify them. As in human sarcomas, immune cells are usually found in infiltrate, staining of mast cells in TMA would have introduced a bias of sampling. This analysis was, then, not performed. However, genes down-expressed by *ATRX*-altered poorly differentiated pleomorphic sarcomas are mostly linked to adaptive immune cell activation, so adaptive immune cells are either less present or less active. This could be achieved by avoiding the release of chemoattractants, and hence, the recruitment or activation of other immune cells. The precise mechanism involved in ATRX loss that changes the immune microenvironment of sarcomas needs to be deciphered.

Regarding *ATRX* expression, 27% of cases (17/63) showed no nuclear ATRX protein, which is consistent with the literature [14,31,32,40]. In tumors presenting FS/NS, 87.5% (14/16) exhibited no ATRX protein at all. In these cases, the *ATRX* mRNA level was low, likely meaning that if the truncated protein is expressed (missed by our screening with the C-terminal antibody), it should be very low. Moreover, if truncated proteins are expressed, the lost domains should be the same in all studied sarcoma types, with partial or complete loss of the helicase C-terminal domain in 90% of cases (18/20) and of both helicase domains in 70% of cases (14/20). As the majority of MS mutations occurred in one helicase domain (71.4%, 5/7) and IHC detected a nuclear localization of the protein, a decrease in ATRX enzymatic activity may be hypothesized [41]. Collectively, these results suggest that alterations of *ATRX* preferentially target its enzymatic functions rather than its protein-protein interactions, thus explaining why mutations in *ATRX* partner genes (i.e., *DAXX*, *EZH2, SP100*) are not frequent and not an alternative to *ATRX* alteration in sarcomas. We, thus, hypothesize that, by modifying its chromatin remodeling action, alterations of *ATRX* trigger a specific transcriptomic program that promotes attenuated mast cell recruitment, leading to the observed immune response in models and human tumors.

Our findings show that *ATRX* alterations are quite frequent in pleomorphic sarcomas (close to 25%) and mostly lead to the loss of ATRX. In addition, we demonstrate that *ATRX* alterations are not only associated with ALT phenotype, but also with a lower differentiation in LMS and immune response regulation in poorly differentiated pleomorphic sarcomas, most probably through non-recruitment of mast cells. Currently, most immunotherapies of sarcomas, which target the adaptive immune system and specifically T cells by helping them to recognize tumors, have a low response rate [42]. Indeed, several recent trials have assessed the response to checkpoint inhibitors, which are used to thwart immune system escape by activating CD8+ cytotoxic T cells [42], with an overall response rate not higher than 25% [43,44]. As targeting the adaptive immune system does not work well in sarcomas, some have tried to target the innate immune system, composed of mast cells, dendritic cells, macrophages, natural killers, and granulocytes, by making therapeutic vaccines that rely on the activation of dendritic cells in the presence of predetermined immunogenic antigen [42]. One trial presented 10 out of 23 patients who lived more than one year, whereas others died after around seven months [45], and another one showed a 1-year progression-free survival of 70.6% [46]. Targeting the innate system, such as mast cells might, therefore, lead to a better outcome for sarcoma patients. And it could be further improved by assessing *ATRX* status before testing mast cell-enhancing therapies, as they have been successful in other solid tumors [47]. These therapies enhanced local mast cell degranulation by using IgE antibodies, as proposed by Singer and Jensen-Jarolim [48]. This strategy could be useful in *ATRX^WT^* tumors to enhance the anti-tumoral action of mast cells, and in *ATRX*-altered sarcomas, to enhance mast cell recruitment and activation [49].

## 5. Conclusions

In conclusion, ATRX altered sarcomas represent roughly ¼ of LMS and of poorly differentiated sarcomas. *ATRX* alterations are mostly truncating and have a direct impact on the protein present in the nucleus. When ATRX is absent from tumors, their transcriptomes are modified toward a lower expression of muscle-related genes in LMS and of immune-related genes in poorly differentiated sarcomas. In cell models of the latter group, ATRX low expression is linked to a higher aggressiveness of tumors and a lower presence of mast cells in tumors. This low percentage could be the first step to the decrease of immune-related genes. This effect of ATRX alteration on the immune system could be used to develop new effective immunotherapies.

## Figures and Tables

**Table 1:** Clinical characteristics in both cohorts, related to Figures 1 and 3. LMS, leiomyosarcoma; UPS, undifferentiated pleomorphic sarcoma; MFS, myxofibrosarcoma; DDLPS, dedifferentiated liposarcoma.

	Cohort1 (*n* = 67)	Cohort2 (*n* = 60)
Follow-up (years)		
Median	5.22	5.94
Range	1.48–19.77	0.01–28.88
Age at diagnosis (years)		
Mean	62.94	62.95
Median	64	64.5
Range	22–80	20–87
Gender		
Female	53 (78.46%)	22 (36.67%)
Male	14 (21.54%)	38 (63.33%)
Tumor site		
Internal trunk	38	9
Uterine	8	0
Member and Trunk wall	21	51
Tumor depth		
Deep	56	42
Superficial	7	5
Superficial and deep	4	13
Tumor size (cm)		
Median	8	8
Range	1.5–23	1–30
Histotype		
LMS	67	0
UPS	0	30
MFS	0	17
DDLPS	0	13
FNCLCC Grade		
I	12	3
II	23	17
III	32	39
Unknown	0	1
Resection status, margins		
R0	42	24
R1	18	28
R2	1	2
Unknown	6	6

## Data Availability

Publicly available datasets were analyzed in this study. This data can be found here: https://dcc.icgc.org/projects/LMS-FR. The other data presented in this study are openly available in Gene Expression Omnibus, reference number GSE71121 (human data) or GSE157953 (mouse data).

## Supplementary Materials

The following are available online at https://www.mdpi.com/article/10.3390/cancers13092151/s1, Figure S1: Multiple correspondence analysis, Figure S2: Example of ATRX staining by immunohistochemistry, Figure S3: ALT status determined by PML/TERF2 immunofluorescence analysis, Figure S4: Kaplan-Meier analysis in leiomyosarcomas (Cohort 1), according to *ATRX* status (wild-type vs. altered), *ATRX* expression (high vs. low), ATRX localization (nucleus vs. absent) and ALT mechanism phenotype (ALT− vs. ALT+), Figure S5: Clustering with differentially expressed genes according to ATRX status, Figure S6: Kaplan-Meier analyses, Figure S7: shRNA against ATRX effect on sarcoma cell lines, Table S1: *ATRX* mutations and structural variants in both cohorts, Table S2: *ATRX* breakpoints in cohort 1, Table S3: *ATRX* fusion transcripts in cohort 2, Table S4: Genomic DNA primers used for point mutations and breakpoint validation, Table S5: Primers used for *ATRX* cDNA screening and validation of mutations, Table S6: Combination of primers used to detect fusion transcript in cohort 2, Supplementary methods.
