# Supplementary material for: ATRX Alteration Contributes to Tumor Growth and Immune Escape in Pleomorphic Sarcomas
Source: Cancers (Basel). 2021 Apr 29;13(9):2151. doi: 10.3390/cancers13092151 (PMC8124877; doi:10.3390/cancers13092151)
Supplement: Supplementary file 1 [file cancers-13-02151-s001.zip › cancers-1174802-supplementary.pdf]

Supplementary figures and legends:

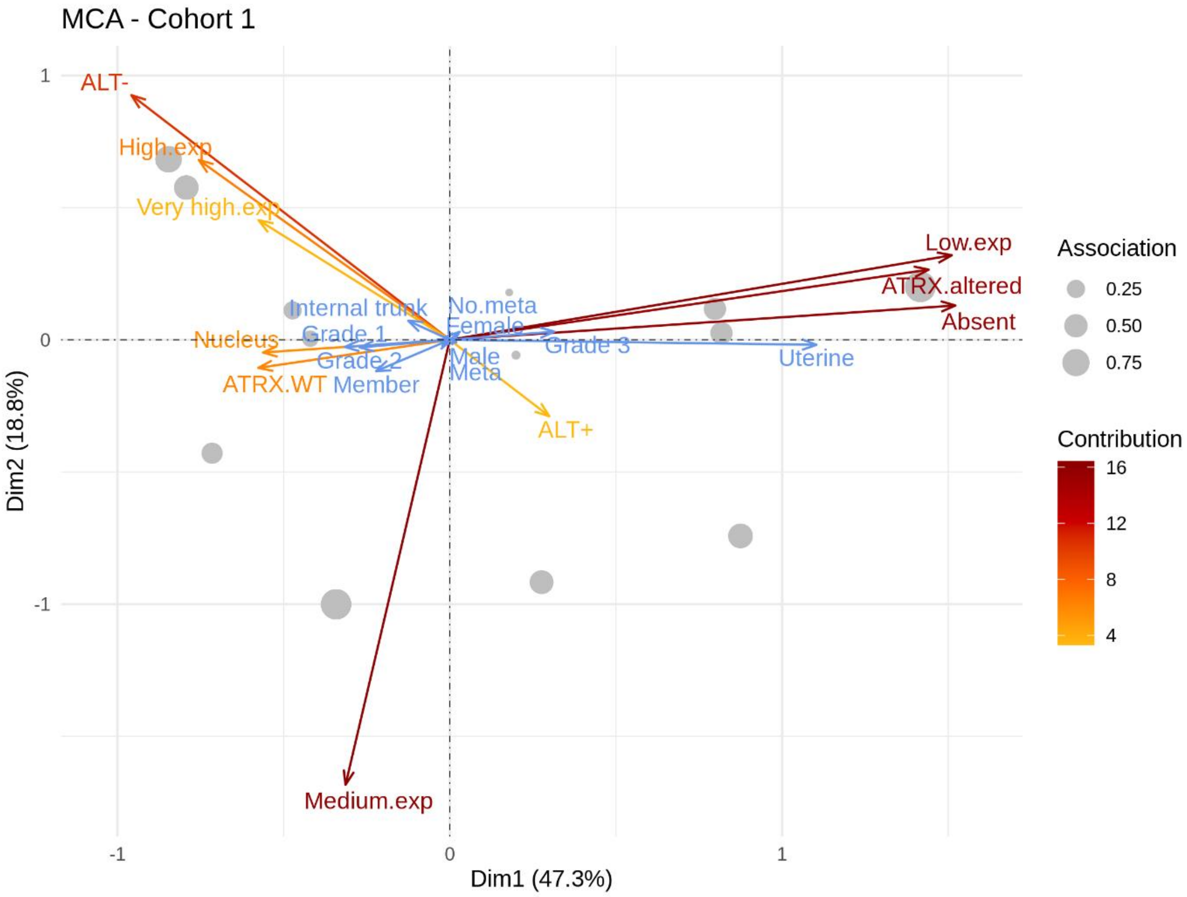

Figure S1: Multiple correspondence analysis related to Figure 2

Multiple correspondence analysis (MCA) was performed on *ATRX* status, mRNA expression, ATRX location and ALT phenotype. Additional variables and sample groups are represented in blue arrows and gray points, respectively.

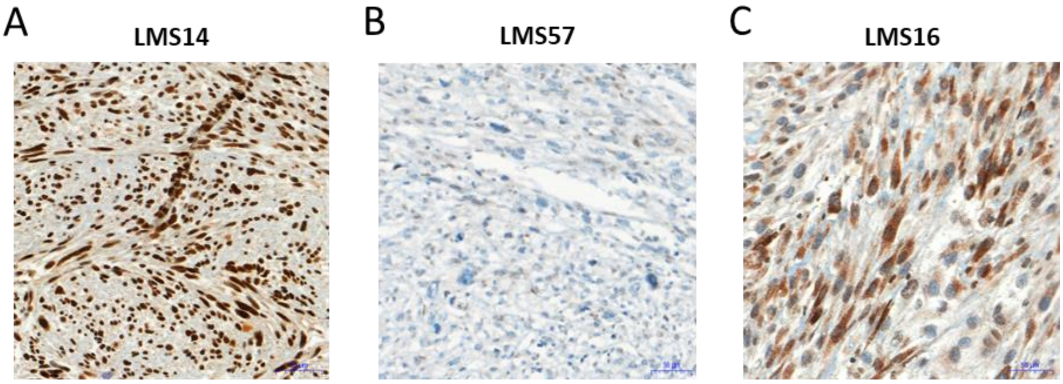

Figure S2: Example of ATRX staining by immunohistochemistry related to Figure 2

Leiomyosarcomas with **A** strong nuclear staining, **B** no tumorous staining or **C** cytoplasmic staining. In all samples, non-tumorous cells serve as internal control and have nuclear staining.

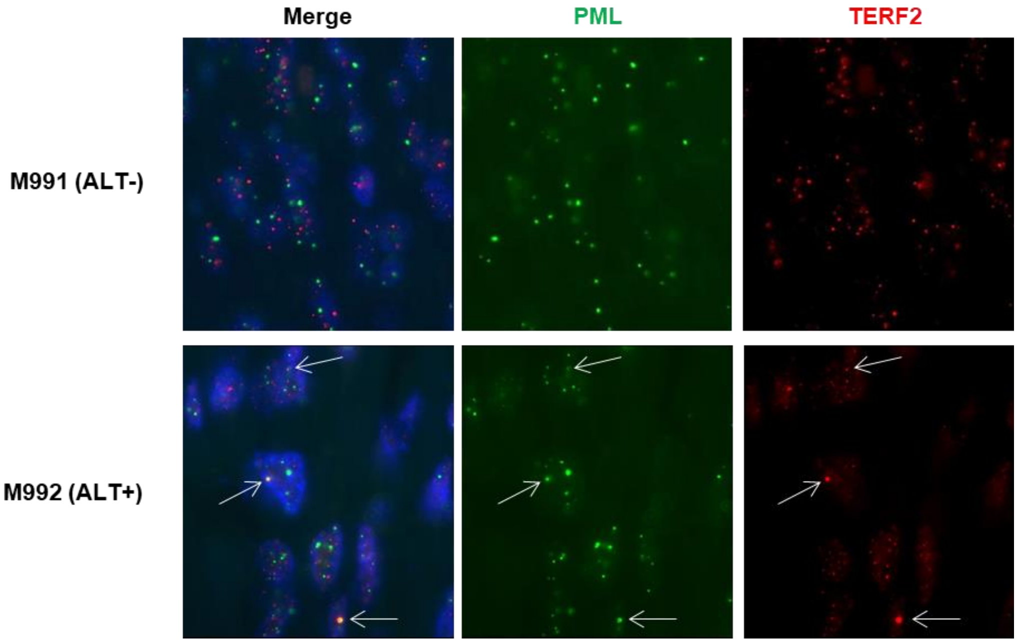

Figure S3: ALT status determined by PML/TERF2 immunofluorescence analysis related to Figure 2

Immunofluorescence showing absence of co-localization of PML (green) and TERF2 (red) signals in a tumor sample (ALT-) and of PML and TERF2 protein co-localization (ALT+), indicating that the ALT mechanism should be active in this tumor (ALT+). Protein co-localizations are highlighted by arrows. Magnification: X1000.

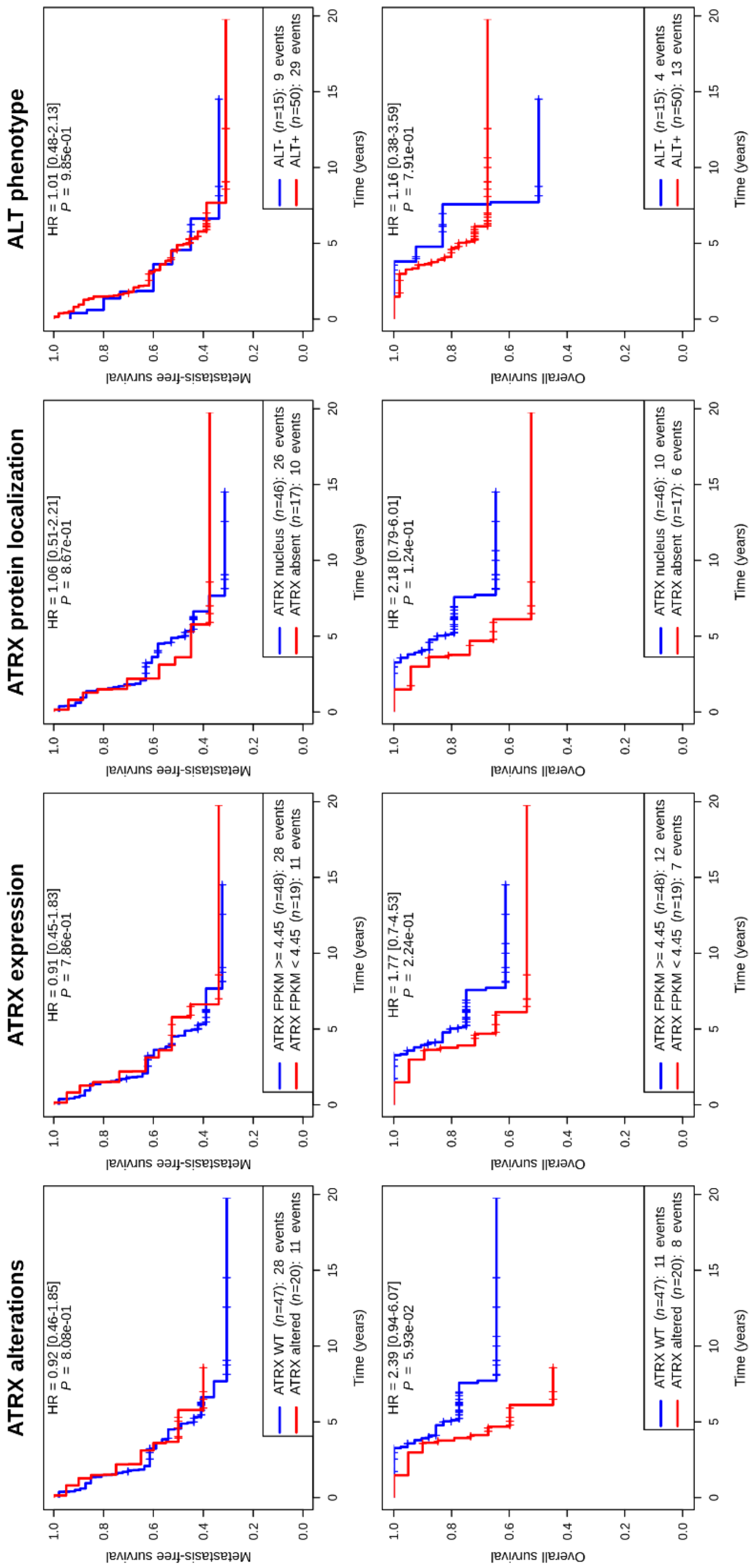

Figure S4: Kaplan-Meier analysis in leiomyosarcomas (Cohort 1), according to *ATRX* status (wild-type vs altered), *ATRX* expression (high *vs* low), ATRX localization (nucleus *vs* absent) and ALT mechanism phenotype (ALT- *vs* ALT+). Up: metastasis-free survival. Down: overall survival. To subdivide *ATRX* expression into two groups, it is plotted for *ATRX* wild-type and altered cases, separately. Intersection between these two density curves is 4.45 (log2(FPKM+1)) related to Figure 2.

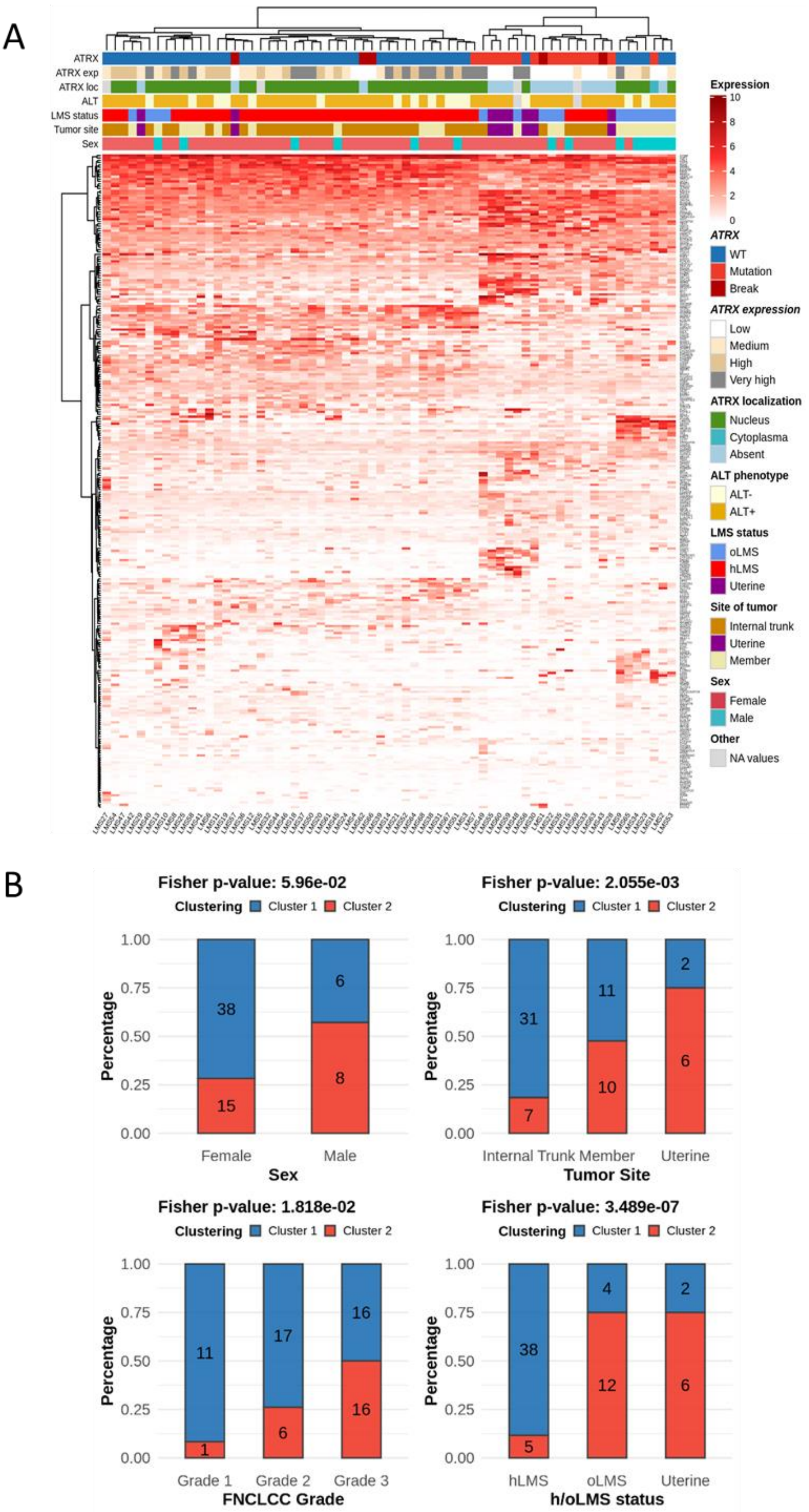

Figure S5: Clustering with differentially expressed genes according to ATRX status related to Figure 3

A mRNA expression (log2(FPKM+1)) of 279 differentially expressed genes in *ATRX* altered tumors (see also Figure 3A; $P \leq 0.01$ and fold-change ≤ -2 or ≥ 2). **B** Association between cluster 1 or 2 (left and right cluster, respectively) to sex, tumor site, grade and LMS status, oLMS and hLMS means “other” and “homogeneous” LMS, respectively (Darbo *et al*, 2020).

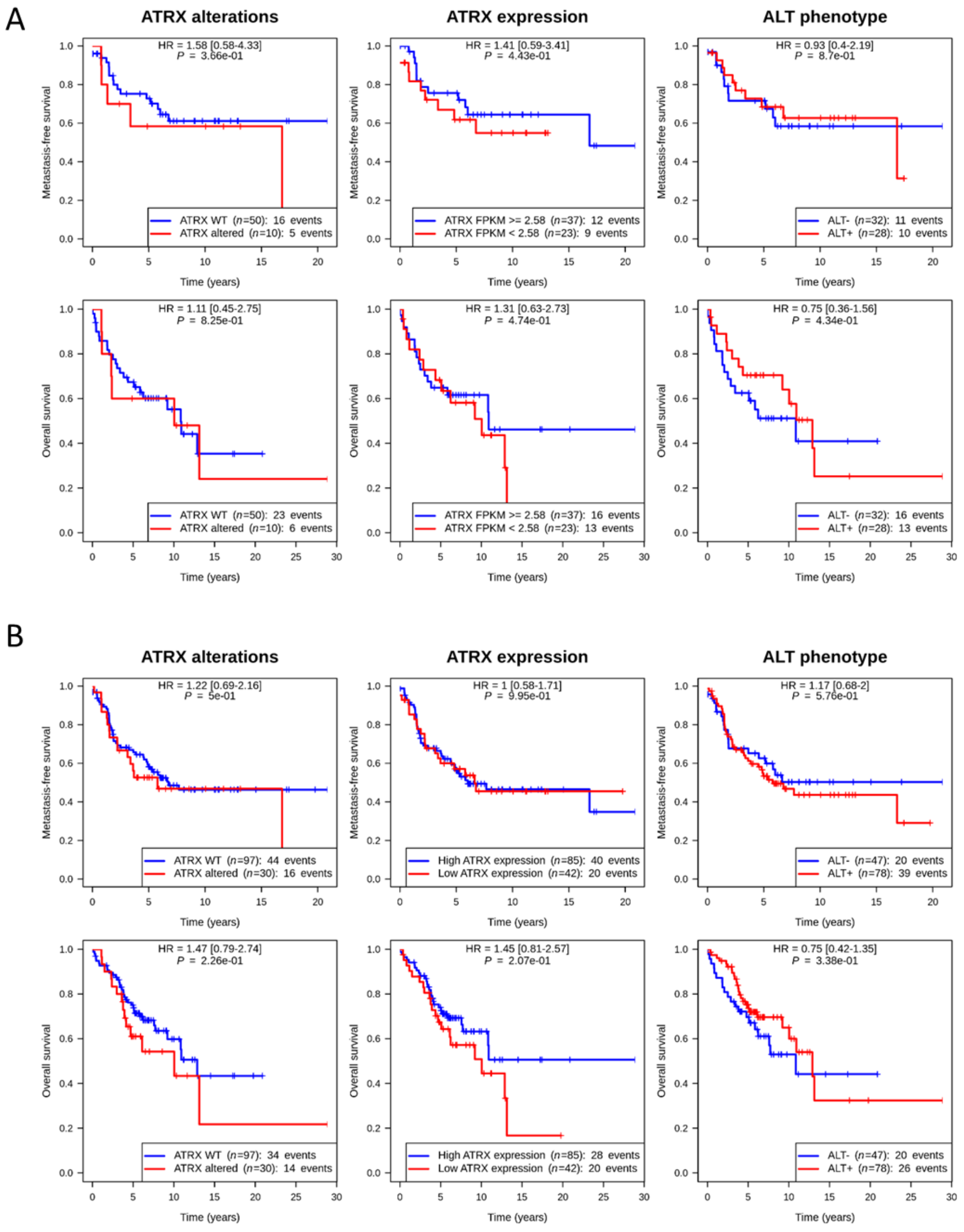

Figure S6: Kaplan-Meier analyses related to Figure 4.

A Kaplan-Meier analysis in poorly differentiated sarcomas (Cohort 2) according to *ATRX* status (wild-type *vs* altered), *ATRX* expression (high *vs* low) and ALT phenotype (ALT- *vs* ALT+). Up: metastasis-free survival. Down: overall survival. To subdivide *ATRX* expression into two groups, it is plotted for *ATRX* wild-type and altered cases separately. Intersection between these two density curves is 2.58 (log2(FPKM+1)). **B** Kaplan-Meier analysis in leiomyosarcomas and poorly differentiated sarcomas (Cohorts 1 and 2), depending on *ATRX* status (wild-type *vs* altered), *ATRX* expression (high *vs* low) and ALT mechanism phenotype (ALT- *vs* ALT+). Up: metastasis-free survival. Down: overall survival. To subdivide *ATRX* expression into two groups, the two cohorts are taken separately to identify the cut-off (see also Supp. Figures 4A and 6A).

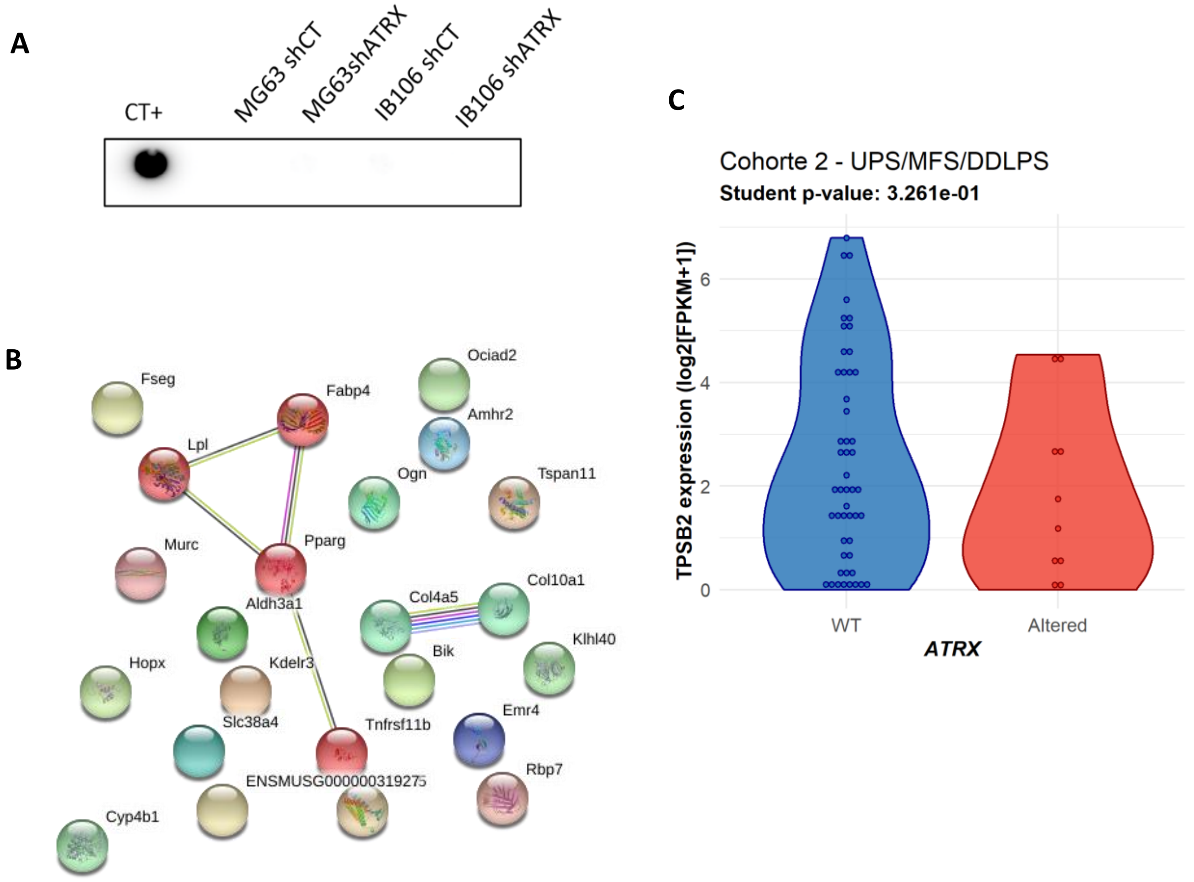

Figure S7: shRNA against ATRX effect on sarcoma cell lines related to Figure 6 and 7.

A C-circle analysis of ALT status upon shRNA against *ATRX* or in control condition. Positive control is U2OS DNA. **B** Links between proteins encoded by genes overexpressed in K7M2 *ATRX*^{KD} tumors displayed by STRING Database showing two clusters via MCL cluster. **C** TPSB2 mRNA expression in log2(FPKM+1) according to ATRX status in cohort 2.

Supplementary tables and legends:

Cohort	Sample	Histotype	Location	Sex	Allele 1	Allele 2	DNA (WGseq) Reads alt/total	DNA (WGseq) Mut. allele freq.	RNA (RNAseq) Reads alt/total	RNA (RNAseq) Mut. exp. freq.	RNA (RNAseq) *ATRX:* log2 (FPKM+1)	Functional *ATRX* status	gDNA (hg19)	cDNA (NM_000489.6)	Protein
1	LMS16T	LMS	Member	M	Frameshift	Y	8/12	0,67	25/34	0,74	3,61	0	chrX: g.76874277_76874281del	c.5441_5445del	p.(C1814SfsX45)
1	LMS22T	LMS	Member	M	Frameshift	Y	11/17	0,65	18/36	0,50	4,10	0	chrX: g.76875903_76875923delinsAAAT	c.5212_5232delinsTTTA	p.(I1738FfsX11)
1	LMS35T	LMS	Member	F	Frameshift		21/59	0,36	19/46	0,41	4,06	1/2	chrX: g.76939939dupG	c.807dupC(;)485_487del	p.(E271RfsX11)(;)(E162del)
1	LMS33T	LMS	Internal Trunk	F	Frameshift		32/61	0,52	109/131	0,83	4,07	0	chrX: g.76938713_76938734del	c.2014_2035del	p.(P672KfsX17)
1	LMS63T	LMS	Internal Trunk	F	Frameshift		28/52	0,54	39/56	0,70	3,99	0	chrX: g.76938581_76938618dup	c.2130_2167dup	p.(E723VfsX26)
1	LMS59T	LMS	Uterus	F	Frameshift		12/52	0,23	34/37	0,92	3,09	0	chrX: g.76938870delT	c.1878delA	p.(K626NfsX23)
1	LMS60T	LMS	Uterus	F	Frameshift		30/54	0,56	4/5	0,80	3,84	0	chrX: g.76849230delG	c.6046delC	p.(H2016NfsX34)
1	LMS15T	LMS	Internal Trunk	M	Nonsense	Y	22/27	0,81	18/25	0,72	3,86	0	chrX: g.76778786C>A	c.6793G>T	p.(E2265X)
1	LMS28T	LMS	Uterus	F	Nonsense		17/47	0,36	44/48	0,92	4,42	0	chrX: g.76889056C>A	c.4954G>T	p.(E1652X)
1	LMS30T	LMS	Uterus	F	Nonsense		20/44	0,45	34/38	0,89	4,14	0	chrX: g.76938583G>C	c.2165C>G	p.(S722X)
1	LMS55T	LMS	Uterus	F	Nonsense		18/44	0,41	18/18	1,00	4,07	0	chrX: g.76907651G>A	c.4510C>T	p.(R1504X)
1	LMS48T	LMS	Member	F	Missense		32/67	0,48	0/66	0,00	5,23	1	chrX: g.76849167C>T	c.6109G>A	p.(V2037I)
1	LMS49T	LMS	Internal Trunk	F	Missense	Isodisomy	41/45	0,91	118/125	0,94	5,51	0	chrX: g.76875980C>T	c.5155G>A	p.(D1719N)
1	LMS69T	LMS	Internal Trunk	F	Missense	Loss	23/28	0,82	59/61	0,97	4,49	0	chrX: g.76890104C>T	c.4790G>A	p.(G1597D)
1	LMS7T	LMS	Internal Trunk	F	Missense		18/51	0,35	95/111	0,86	5,19	0	chrX: g.76890113T>C	c.4781A>G	p.(H1594R)
1	LMS57T	LMS	Uterus	F	Del. & FS		NA	NA	39/49	0,80	4,36	0	chrX: g.76882435_76982612del	c.21_4956del	p.(S7RfsX3)
1	LMS66T	LMS	Internal Trunk	F	Del. & FS		NA	NA	45/48	0,94	3,85	0	chrX:g.76756028_76920645delinsATTCCA	c.3810_7479delins3809+1_3809+187	p.(I1271YfsX62)
1	LMS1T	LMS	Internal Trunk	F	Del. Inv. Ins.		NA	NA	NA	NA	2,55	0	chrX: g.76961353_77042805delins77042774_77042825inv	NA	NA
1	LMS43T	LMS	Internal Trunk	F	Del.		NA	NA	NA	NA	1,91	0	chrX: g.77000959_77060888del	NA	NA
1	LMS62T	LMS	Internal Trunk	F	Trans. Complex		NA	NA	NA	NA	2,22	0	Complex	NA	NA

Cohort	Sample	Histotype	Location	Sex	Allele 1	Allele 2	Sanger DNA allelic status	Reads alt/total	Mut. exp. freq.	*ATRX:* log2 (FPKM+1)	Functional *ATRX* status	gDNA (hg19)	cDNA (NM_000489.6)	Protein
2	S873	MFS	Member	M	Frameshift	Y	B	0/4*	Sanger: B	2,39	0	g.76874280_76874290del	c.5432_5442del	p.(L1811CfsX46)
2	N003	DDLPS	Internal Trunk	M	Nonsense	Y	A/B (heterogenous)	0/3*	Sanger: A/B	0,86	0	g.76938583G>C	c.2165C>G	p.(Ser722X)
2	S859	UPS	Member	M	Nonsense	Y	B	8/8	1,00	2,64	0	g.76875866C>A	c.5269G>T	p.(E1757X)
2	S825	UPS	Trunk wall	M	Missense	Y	B	21/21	1,00	3,24	0	g.76938098C>T	c.2650G>A	p.(E884K)
2	S928	UPS	Member	F	Missense		A/B	8/8	1,00	2,67	0	g.76875938T>C	c.5197A>G	p.(K1733E)
2	S832	MFS	Member	F	Missense		A/B	6/6	1,00	2,90	0	g.76938571T>A	c.2177A>T	p.(D726V)
2	S823	MFS	Member	M	Fusion transcript	Y	NA	NA	NA	0,98	0	NA	NA	p.(S7RfsX62)
2	S831	UPS	Trunk wall	M	Fusion transcript	Y	NA	NA	NA	2,47	0	NA	NA	p.(D2234NfsX32;D2234RfsX4)
2	S918	UPS	Member	F	Fusion transcript		NA	NA	NA	2,16	0	NA	NA	p.(V1520DfsX54;I1521VfsX33)
2	M979	MFS	Member	F	Fusion transcript		NA	NA	NA	2,04	0	NA	NA	p.(R2109SfsX12)

Table S1: *ATRX* mutations and structural variants in both cohorts related to Figure 2 and 4.

For each of the 30 validated alterations of *ATRX* (NM_000489.6), genomic and expression information and annotations are reported in the table. Regarding cohort 2, only gDNA Sanger sequencing data for point mutations are available. *: not detected by RNAseq but detected by Sanger sequencing on cDNA. Mutation expression frequency for these two mutations could not be determined using RNAseq, probably because of tumor heterogeneity, so allele expression in RNA was defined according to Sanger sequencing data. Alt: alternative, Freq.: frequency, Exp.: expression, Mut.: mutation, Loc.: location, M: Male, F: Female, LMS: leiomyosarcoma, DDLPS: dedifferentiated liposarcoma, MFS: myxofibrosarcoma, UPS: undifferentiated pleomorphic sarcoma, Y: chromosome Y, A: reference allele, B: alternative allele, Del: deletion, Ins: insertion, Inv: inversion, FS: Frameshift, SV: structural variant, Trans.: translocation, FPKM: Fragments Per Kilobase Million, NA: not available.

Sample	Histotype	Sex	Gene_chromosome1	Break location	Break position (hg19)	Gene_chromosome2	Gene name 2	Break location 2	Break position 2 (hg19)	Mechanism	Consequence on *ATRX* gene	Genomic nomenclature (hg19) chrX	cDNA nomenclature (NM_000489.6)	Protein nomenclature
			ATRX			Fusion partner				Annotations				
LMS1T	LMS	F	X	Intron 2	76961352	X	**Intergenic**	X	77042825	Deletion/inversion-insertion	Loss of 3' part to exon 2	g.76961353_77042805delins77042774_77042825inv	-	-
LMS43T	LMS	F	X	Intron 1	77000958	X	**Intergenic**	X	77060889	Deletion	Loss of 3' part to exon 1	g.77000959_77060888del	-	-
LMS57T	LMS	F	X	Intron 19	76882434	X	***ATRX***	Intron 1	76982613	Deletion	Fusion of intron 1 to intron 19	g.76882435_76982612del	c.21_4956del	p.(S7RfsX3)
LMS62T	LMS	F	X	Intron 1	77035789	X	***DMD***	Intron 60	31367514	Complex	Loss of 3' part to exon 1	Complex	-	-
LMS66T	LMS	F	X	Intron 10	76920646	X	**Intergenic**	X	76756027	Deletion	Loss of exon 11 to the end	g.76756028_76920645delinsATTCCA	c.3810_7479delins3809+1_3809+187	p.(I1271YfsX62)

Table S2: *ATRX* breakpoints in cohort 1 related to Figure 1.

Chromosomal and genomic coordinates of gene breakpoint regarding *ATRX* and its fusion partners are indicated together with the potential mechanism involved and consequence for the *ATRX* gene. Nomenclatures of the alteration and its effect on RNA are indicated when an alternative RNA product was detected by RNAseq. Predicted effect on protein is then indicated. M: Male, F: Female, LMS: leiomyosarcoma.

Sample	Histotype	Sex	*ATRX*			Fusion partner				Annotations		
			Gene_chromosome1	Break location	Break position (hg19)	Gene_chromosome2	Gene name 2	Break location 2	Break position 2 (hg19)	Mechanism	ORF conservation	ATRX Protein
S823	MFS	M	X	exon 1	77041470	15	***DDX11L9***	exon 1	102519162	Translocation	No	p.(S7RfsX62)
S831	UPS	M	X	exon 30	76812922	X	***RNU6-974P***	downstream	81166026	Eversion	No	p.(D2234NfsX32;D2234RfsX4)
S918	UPS	F	X	exon 15	76891547	X	***RP1-279N11.1***	upstream	75878708	Deletion	No	p.(V1520DfsX54;I1521VfsX33)
M979	MFS	F	X	exon 28	76829715	X	***FTX***	intron	73401759	Inversion	No	p.(R2109SfsX12)

Table S3: *ATRX* fusion transcripts in cohort 2 related to Figure 4.

Chromosomal and genomic coordinates of gene breakpoint regarding *ATRX* and its fusion partners are indicated together with the potential mechanism involved and predicted effect on the protein. MFS: myxofibrosarcoma, UPS: undifferentiated pleomorphic sarcoma, M: Male, F: Female.

Primer name	Primer sequence 5'-->3'	Location	Product size (bp)	PCR program	Ref gene	Tumor
ATRXgDNAex7F	ACTTGTGTCCAATATGCCATTTT	Exon 7	366	TD 60°C	NM_000489.6	LMS35T
ATRXgDNAex7R	AGAAGTCTTCCAAGGGCAGA					
ATRXgDNAex9F	GCGTAATTCTTCTGACAGTGCT	Exon 9	346	TD 60°C		N003/ LMS35T
ATRXgDNAex9R	AGAAGACTCAGACTGGGTTTGT					
ATRXgDNAex17F	TGCTGTTTCTTAGAAGTTTTGGTT	Exon 17	485	TD 60°C		M987
ATRXgDNAex17R	CATTAGGACCTCTGCTCAAACA					
ATRXgDNAex20F	CAACGATGTCATTTTATCTTCCTG	Exon 20	452	TD 60°C		S928/S859/LMS22T
ATRXgDNAex20R	ACCACTCATTTATAAAGCATCTCA					
ATRXgDNAex21F	TGAGCATTTCATTGGGGAAT	Exon 21	326	TD 60°C		S873/LMS16T
ATRXgDNAex21R	GCTCAGAAAATATGTTGGGATTG					
ATRXgDNAex26F	CTCCCAAGTCCCCATCAGTT	Exon 26	377	TD 60°C		LMS48T/LMS60T
ATRXgDNAex26R	AGGAAGGAAGGAAAAGCAACA					
ATRXBPLMS1F1	AACTGGCAATCAAGTCTGTGC	*ATRX* intron 2	268	TD 60°C		LMS1T
ATRXBPLMS1R1	TCCAAAATTCATATGGACCAGGC					
ATRXBPLMS1F2	GAGGTCGAGGTTGGAGGATG	Upstream *ATRX* gene	153	TD 60°C		
ATRXBPLMS1R2	CACCTCAGCCTCTCAAGCAG					
ATRXBPLMS43F1	CGTCCTAGCCTCTGGTAACC	*ATRX* intron 1	206	TD 60°C		LMS43T
ATRXBPLMS43R1	TGGAGTCCTATTCAGCCCTT					
ATRXBPLMS43F2	AATGTCTTTCTGTGCCTGGC	Intergenic chrX	182	TD 60°C		
ATRXBPLMS43R2	GGCAGAAGGATCGCTTGAAC					
ATRXBPLMS57F1	TTCACCGTGTTAGCCAGGAT	*ATRX* intron 19	390	TD 60°C		LMS57T
ATRXBPLMS57R1	ACCACCTAAATGTTGCAATACCA					
ATRXBPLMS57F2	TCCACATCCTCTCCAGCATC	*ATRX* intron 1	476	TD 60°C		
ATRXBPLMS57R2	GCCTATCTGCAATTGGGTCA					
ATRXBPLMS62F1	AGCCTTGCCTGTACTTCTTTG	*DMD* intron 60	151	TD 60°C		LMS62T
ATRXBPLMS62R1	CATGCCTGTAGTCCCAGCT					
ATRXBPLMS62F2	GGCGACAGGGTGAGAGTC	*ATRX* intron 1	206	TD 60°C		
ATRXBPLMS62R2	AGCAACAAAACACCTGTAACCT					
ATRXBPLMS66F1	GGGAATTGAACAATGAGAACACA	Intergenic chrX	118	TD 60°C		LMS66T
ATRXBPLMS66R1	GTTTGCCGCACCTACTGAC					
ATRXBPLMS66F2	TGCTGTTGAATAAAACCTCTCGT	*ATRX* intron 10	150	TD 60°C		
ATRXBPLMS66R2	TTTGATGCTGCATAACCTTCCA					

Table S4: Genomic DNA primers used for point mutations and breakpoint validation by Sanger sequencing related to methods. Forward and reverse primers used for mutation and breakpoint (BP) validations on genomic DNA are presented. All mutations were detected by two independent techniques and only those not detected by both WGseq and RNAseq were validated by Sanger sequencing. RNA primers were used for DNA validation of some *ATRX* mutations in exon 9, see Supplementary Table 6. For each BP validation in cohort 1, the two primers used to detect the BP are in red: other primers were used to detect a potential reciprocal translocation. PCR was a touch-down 60°C program (TD 60°C) (2 cycles at a temperature of 60°C, followed by 2 cycles at 59°C, 2 cycles at 58°C, 3 cycles at 57°C, 3 cycles at 56°C, 4 cycles at 55°C, 4 cycles at 54°C, 5 cycles at 53°C and finally 10 cycles at 52°C). RefSeq annotation used to locate primers for the gene is indicated. Tumors in which each primer couple was used are indicated. Ex: exon, bp: base pair, F: forward, R: reverse.

Primer name	Primer sequence 5'-->3'	Product size (bp)	DNA validation	RNA validation
ATRXcDNAF1	ACCGCTGAGCCCATGAGT	143		
ATRXcDNAR1	CCACTGATTTTATCTGTGTTTTGA			
ATRXcDNAF2	TTCCTTGCACACTCATCAGAA	399		
ATRXcDNAR2	TTTCATTTTACTTCTGCTTCTAAATTC			
ATRXcDNAF3	CAGAGCCAGTGCTGAATGAA	627		LMS35T
ATRXcDNAR3	CAGAGCCAGAACAGGAATCA			
ATRXcDNAF4	CAGTTGTTGCAGCAAAATAAGAA	495		
ATRXcDNAR4	TTCCAAAGCACAAGGTTTTTC			
ATRXcDNAF5	TGGATGCTGTAAACAAAGAGAAA	552		
ATRXcDNAR5	CAGCACCTTTAATTGGGGAAT			
ATRXcDNAF6	GGAGGTATTAAATCAAAAACTACAGC	579	LMS33T/LMS59T LMS63T/S832	LMS59T/LMS63T
ATRXcDNAR6	CAAAGTCTTATGGTTTGTATGAATTT			
ATRXcDNAF7	TGAAATGCTAGCAATCCTCAAA	773	S825	
ATRXcDNAR7	CAGTTCCCTTTTTGCTCTGC			
ATRXcDNAF8	AAGTACAGGATGGCTTATCTGATATT	699		
ATRXcDNAR8	GGGAGTTTCTCTTTTTCTCCTTG			
ATRXcDNAF9	CAAAGTGGCTCATCATCATCTG	684		
ATRXcDNAR9	TTCACTGCTCACCTTTCTTCTG			
ATRXcDNAF10	TGAGTGACGGAGAATCTGGA	696		
ATRXcDNAR10	CAGACTCACAGCAGCAATCC			
ATRXcDNAF11	AAGATGCTTCACCCACCAAG	802		LMS22T
ATRXcDNAR11	TCTGCACACTGACCATTTTGA			
ATRXcDNAF12	CATTGTATGGTTAATTTTATCAAGGAA	761		LMS16T
ATRXcDNAR12	TCAGCATCAGCATCTGTAACAA			
ATRXcDNAF13	TTTAAAACTGGAAGAAAGTAAAGCTAC	741		LMS48T/LMS60T
ATRXcDNAR13	TCCCTCTTCTTCTTCTTTTCTGA			
ATRXcDNAF14	TGGAGCGTCATTTTACTATGAAT	559		
ATRXcDNAR14	TCCTGGCTGGCTTGTCTACT			
ATRXcDNAF15	ACAGTGTGACAGCAGTGAGGA	545		
ATRXcDNAR15	TTGAGTTCTGTTAAGTCATTGATTCC			
ATRXcDNALMS33F	GTGGACTTGGACAGGAAAACA	690		LMS33T
ATRXcDNALMS33R	TGCTGTGTTTCTCATCTTCAGA			
ATRXcDNALMS35F	AACAATGTAGGTGGTGTGCG	234		LMS35T
ATRXcDNALMS35R	TCTTATTTTGCTGCAACAACTGT			
ATRXBPcDNALMS66F1	TCATCCTCTAGTTTGAAGCAAGG	233		LMS66T
ATRXBPcDNALMS66R2	CCTCAGCAAACTAACACAGG			

Table S5: Primers used for *ATRX* cDNA screening and validation of mutations by Sanger sequencing related to methods.

ATRX forward and reverse primers used in cohort 2 for *ATRX* cDNA screening in both cohorts for some exon 9 *ATRX* DNA mutations and in cohort 1 for validation of cDNA mutations are presented. TD60°C PCR program described in Supplementary Table 5 legend was used. Primers used for cDNA validation of fusion transcript in LMS66 are also presented (primers in red). Tumors of cohort 1 in which each primer couple was used are indicated. For chohort 2 primers numbered from 1 to 15 were all used in all tumors to performed the ATRX RNA screen. bp: base pair, F: forward, R: reverse.

Primer name	Primer sequence 5'-->3'	Tumor
ATRXex1F	CAGTGCATTTCTATCGTAACCG	S823
DDX11L9ex1R	CTCTAGGCATGGCTCCTCTC	
ATRXex15F	TGAGAACAGAAACACAAAATGCT	S918
RP1-279N11.1 75878708F	CACAACTGGCTCAACTGCTT	
ATRXex15F	TGAGAACAGAAACACAAAATGCT	
RP1-279N11.1 75879066F	TCATGTCAGTCTTTGTTGAGCA	
ATRXex27F	GTCCCTCATATCTCTGGACTTGA	
RP1-279N11.1 75913486F	TCTCTGTCCAGGCTTTCTTGA	
ATRXex27F	GTCCCTCATATCTCTGGACTTGA	M979
FTXintronF	TGGAAGAATGTAAGGCCAGG	
ATRXex30F	CAGGTGGAGCGTCATTTTACTA	S831
RNU6-974P81233619R	CAAGAATGTGGAGAGAGCCTTC	
ATRXex30F	CAGGTGGAGCGTCATTTTACTA	
RNU6-974P81166026R	GCAGTTGACACTTGCTACAATG	
DDX11L9ex1F	CTCTTAGCCCAGACTTCCCG	S823
ATRXex1R	CAGAGTTACTTCCAGAACCACT	
RP1-279N11.1 75913486R	AGTGCTCTCTCTTGTTTATGGGA	S918
ATRXex27R	CTCGATTAGCAGCTACCAGAT	
RP1-279N11.1 75878708R	TCAACTTTGCTGAGCCAAACT	
ATRXex15R	TCTTACCAAGGCCCATACAGT	
RP1-279N11.1 75879066R	GCCCTGGAGCTTACAATCAG	
ATRXex15R	TCTTACCAAGGCCCATACAGT	
FTXintronR	CAGGGAGCTTTCACATCAAACA	M979
ATRXex27R	CTCGATTAGCAGCTACCAGAT	
RNU6-974P81233619F	TCACTGAGAACATTCAACCCC	S831
ATRXex30R	GTTGATTACTCATTGCTGACAGG	
RNU6-974P81166026F	GAGGAAGACAAGTTTGGGCA	
ATRXex30R	GTTGATTACTCATTGCTGACAGG	

Table S6: Combination of primers used to detect fusion transcript in cohort 2 related to methods.

First part of table indicates primer combination used to detect each fusion transcript. Second part describes primer combination used to detect potential reciprocal fusion transcript. TD60°C PCR program described in Supplementary Table 5 legend was used. F: forward, R: reverse, Ex: exon.

Supplementary methods:

DNA extraction

For both cohorts, genomic DNA from frozen samples was isolated using standard phenol-chloroform extraction protocol. DNA was quantified using Nanodrop 1000 spectrophotometer (Thermo Fisher Scientific, Waltham, MA, USA). Blood material was also available for cohort 1. Genomic DNA from blood samples was extracted using customized automated purification of DNA from compromised blood samples on the Autopure LS device according to the manufacturer's recommendations (9001340, Qiagen, Hilden, Germany) with increased centrifugation of 10 min for DNA precipitation and DNA wash.

Whole genome sequencing and analysis

Whole genome sequencing was performed only on cohort 1. To construct short-insert paired-end libraries, a no-PCR protocol was used with the TruSeq™DNA Sample Preparation Kit v2 (FC-121-2001/FC-121-2002, Illumina Inc., San Diego, CA, USA) and the KAPA Library Preparation Kit (KK8235, Kapa Biosystems, Basel, Switzerland). Briefly, 2 µg of genomic DNA were sheared on a Covaris™ E220, size-selected and concentrated using AMPure XP beads (A63880, Agencourt, Beckman Coulter, Brea, CA, USA) in order to reach a fragment size of 220 – 480 bp. Fragmented DNA was end-repaired, adenylated and ligated to Illumina-specific indexed paired-end adapters.

DNA sequencing was performed in paired-end mode, 2x100 bp or 2x125 bp according to flowcell version, in five or three sequencing lanes of HiSeq2000 flowcell v3 or v4 (Illumina Inc., San Diego, CA, USA) to analyze tumor or normal/constitutive samples and to reach minimal yield of 145 or 85 Gb, respectively. Two tumor samples were sequenced in 20 lanes to reach a minimal yield of 560 Gb. Image analysis, base calling and quality scoring of the run were processed using the manufacturer's Real Time Analysis software (RTA 1.13.48) and followed by generation of FASTQ sequence files by CASAVA (Illumina Inc., San Diego, CA, USA).

DNA reads were trimmed of the 5' and 3' low quality bases (phred cut-off 20, max trim size 30 nt) and sequencing adapters were removed with Sickle2 [44] (https://github.com/najoshi/sickle) and SeqPrep3 (https://github.com/jstjohn/SeqPrep), respectively. Then, DNA-curated sequences were aligned using Bowtie v2.2.1.0 [45], with the very sensitive option, on the Human Genome version hg19. Thus, aligned reads were filtered

out if their alignment score was less than 20 or if they were duplicated PCR reads, with SAMtools v0.1.19 [46] and PicardTools v1.118 [47] (http://broadinstitute.github.io/picard/), respectively. SNV were detected by SAMtools mpileup v0.1.19 [46], with a minimum of 20 as phred quality score (-Q 20), and by bcftools call -Am (SAMtools v0.2.0, http://samtools.github.io/bcftools/call-m.pdf).

RNA extraction, sequencing and analysis

For both cohorts, RNA extraction was performed using standard TRIzol (15596026, Thermo Fisher, Waltham, MA, USA) / chloroform extraction (32211-1L, Fisher Scientific, Hampton, NH, USA) followed by 100% ethanol precipitation and RNA purification using the RNeasy Mini Kit (74104, Qiagen, Hilden, Germany) with DNase treatment (79254, RNase-Free DNase Set, Qiagen, Hilden, Germany). Total RNA was quantified using a Nanodrop 1000 spectrophotometer (Thermo Scientific, Waltham, MA, USA) and qualified with an Agilent 2100 Bioanalyzer (Agilent, Santa Clara, CA, USA) using the Agilent RNA 6000 Nano Kit (5067-1511, Agilent, Santa Clara, CA, USA) according to the manufacturer's instructions.

To control the sequencing quality, ERCC RNA Spike-In Mix (4456740, Life technologies, Carlsbad, CA, USA) were added to each RNA sample as recommended by the manufacturer. Analysis of this quality control was performed as previously described [18].

Regarding cohort 1, libraries from total RNA were prepared using the TruSeq®Stranded Total RNA Gold Library Preparation Kit (RS-122-2301, Illumina Inc., San Diego, CA, USA) according to the manufacturer's protocol. Briefly, 0.5 µg of total RNA was ribo-depleted using the Ribo-Zero Gold Kit. RNA fragmentation resulted in fragments of 80 – 450 nt, with a major peak at 160 nt. First-strand cDNA synthesis by random hexamers and reverse transcriptase was followed by second-strand cDNA synthesis, performed in presence of dUTP instead of dTTP. Blunt-ended double-stranded cDNA was 3´adenylated and Illumina-indexed adapters were ligated. Resulting libraries were enriched with 15 PCR cycles.

Construction of cohort 2 libraries was the same as previously described for frozen samples [18].

Libraries, of these two cohorts were sequenced on HiSeq2000 (Illumina Inc., San Diego, CA, USA) in paired-end mode with a read length of 2x75 bp using TruSeq SBS Kit v3-HS (FC-401-3001, Illumina Inc., San Diego, CA, USA). Image analysis, base calling and base quality scoring of the run were processed by integrated primary Real Time Analysis (RTA 1.13.48) software and followed by generation of FASTQ sequence files by CASAVA (v1.8, Illumina

Inc., San Diego, CA, USA). Library construction and RNA sequencing were performed at the Centro Nacional de Análisis Genómico (CNAG, Barcelona, Spain).

RNA bioinformatic analysis (alignment and expression quantification) for these two cohorts was performed as previously described [18]. Fusion transcripts were detected with Defuse v0.6.1 [48] as previously described [49].

Regarding cohort 1, SNV (Single Nucleotide Variant) were detected using samtools mpileup (SAMtools v0.1.19 [46]), with a minimum of 20 as phred quality score (-Q 20), and bcftools view -cvg (SAMtools v0.1.19 [50]). Regarding cohort 2, SNV were detected using samtools mpileup [46], with a minimum of 20 as phred quality score (-Q 20), and bcftools view -cgN [50]. Detected variants with fewer than 5 coverage reads were filtered out.

Annotation of variants

Regarding cohort 1, variants detected in constitutional, tumor DNA and tumor RNA were merged in the same file. Then, somatic variants were extracted with: (i) a minimum coverage of 14 reads in the tumor and 8 in the normal and (ii) a minimal allelic fraction of 0.3 in tumor and 0 in normal.

Since tumor and constitutional DNA were not available for cohort 2, candidate variants could not be filtered based on genotype differences between constitutional DNA and DNA/RNA tumor. Instead, filters applied were the same as for cohort 1 above, but with a minimum coverage of 5 reads due to a lower sequencing depth.

Variants were annotated using the Annovar v20160314 tool [51]. Variants were selected whose alternative allele frequency (AF) in the Caucasian population (CEU) is lower than 0.1%, as reported in the 1000Genome database [52]. Finally, variants were kept if they were localized in coding regions and were non-synonymous.

Break Point Detection

Structural variants (SV) were detected from paired tumor/normal whole genome high-quality sequencing data. Paired-end reads were aligned using Bowtie v2.2.1.0 [45], very sensitive local option allowing soft-clipped sequences. The algorithm has three main steps: i) identification of potential breakpoints, ii) characterization of the second side of the breakpoints, and iii) selection of high-confidence breakpoints. All parameters were set to analyze 60X tumor and 30X normal sequencing depth. Very conservative filters were used to minimize false positive detection.

i) Identification: at this step, reads with at least one soft-clipped end were analyzed as singletons. A position was considered as a potential breakpoint if it was covered by at least 4 soft-clipped reads, 5 soft-clipped bases (with at least two occurrences of two different bases), and if they represented more than 5% of the total amount of reads at this position in the tumor sample. We selected potential somatic events by discarding positions covered by at least 1 read and 1 base in a surrounding 5-nucleotide window in the normal sample. We refer to them as the "first side" of the breakpoint.

ii) Characterization: to determine the genomic positions of the soft-clipped sequence from selected reads, we used the UCSC blat server [53]. If no match was returned, the reverse complement sequence was pulled to test. If there was still no match, the BAM file was investigated for some soft-clip somatic position around the discordant or oversized-insert read mate (hereafter named abnormal) location from the first side of the breakpoint. Because of the small size of the soft-clipped sequence, multiple matches can be found. We used soft-clipped abnormal read mates to select matches with the most coherent chromosomic locations. We refer to them as the "second side" of the breakpoint.

iii) Selection: Positions detected from both the first and second sides (in a 5-nucleotide window) were defined as the common pool. We considered as artifacts (due to repeat regions for instance) couples of positions covered with reads and associated soft-clipped sequences separated by fewer than 15 nucleotides and discarded them. We classified the breakpoints in three groups: high-confidence breakpoints, breakpoints needing investigation, and unique position breakpoints. If a breakpoint was covered by reads and associated soft-clipped sequences having both positions belonging to the common pool, it was classified in the first group. If a breakpoint was covered by reads and associated soft-clipped sequences having only one of the positions belonging to the common pool, it was classified in the second group. Then the missing position was searched among the filtered positions. If it was present in the normal sample, the position was discarded and the breakpoint was completed otherwise. Finally, the third group corresponds to breakpoints with both sides outside the common pool and considered as unique: these were discarded. The sides of breakpoints were sorted according to their chromosomic positions to avoid duplicates.

For all the 67 LMS (cohort 1), the whole *ATRX* sequence obtained by WG sequencing was visualized using the Integrative Genomics Viewer (IGV, v2.6.3) [54] and soft-clipped reads were detected for 5 cases. *ATRX* fusion partner sequences and breakpoints location could be determined thanks to the blat function on the UCSC website.

PCR on genomic DNA

For screening of mutations on genomic DNA, PCR primers were designed using the Primer 3 program (44)(https://bioinfo.ut.ee/primer3-0.4.0/) and are presented in Table S4. All PCR were performed on 50ng of DNA using AmpliTaqGold® DNA polymerase (4311820, Applied Biosystems, Foster City, CA, USA) according to the manufacturer's instructions with the PCR program described in the Table S4 Legend.

RT-PCR

Total RNA was first reverse-transcribed using random hexamers and the High Capacity cDNA Reverse Transcription Kit (4368814, Applied Biosystems, Foster City, CA, USA) according to the manufacturer's instructions. All primers used were designed using the Primer 3 program (44)(https://bioinfo.ut.ee/primer3-0.4.0/). For *ATRX* cDNA screening, primers used are presented in Table S5. For fusion transcript detection, control PCR were first performed with different forward and reverse primers for each gene implicated in the fusion, and then PCR was performed using a forward primer for one gene and reverse primer for the other gene (Table S6). All PCR were performed as previously described for PCR on genomic DNA.

Sanger Sequencing

PCR products were purified using an ExoSAP-IT PCR Purification Kit (US78200, GE Healthcare, Piscataway, NJ, USA) and sequencing reactions were performed with the Big Dye Terminator V1.1 Kit (4336805, Applied Biosystems, Foster City, CA, USA) according to the manufacturer's recommendations. Samples were purified using the Big Dye XTerminator Purification kit (4376486, Applied Biosystems, Foster City, CA, USA) according to the manufacturer's instructions and sequencing was performed on a 3730xl Genetic Analyzer for cohort 1 or 3130xl Genetic Analyzer for cohort 2 (Applied Biosystems, Foster City, CA, USA). Sequences were then analyzed using the Sequencing analysis V5.3.1 and the SeqScape V2.6 software (Life Technologies, Carlsbad, CA, USA). FinchTV software (V1.4.0) was also used (Geospiza, Seattle, WA, USA).

Immunohistochemistry

Sixty-seven tumors in cohort 1 were analyzed on tissue microarrays. Each case was represented by three spots 4-µm-thick and 1mm in diameter. Immunohistochemistry was performed on a BenchMark Ultra instrument (Ventana, Washington D.C, USA). Antigen retrieval was performed using a CC1 protocol for 16 min at 98°C (Ventana, Washington D.C, USA), and the

anti-ATRX antibody (1:1000, BSB3297, Clone BSB-108, Diagomics, Blagnac, France) was diluted in PREPKIT9 for 20 min. Antibody detection was performed using the Optiview detection kit (860-099, Ventana, Washington D.C, USA). Immunohistochemical pictures were taken using a Panoramic 250 Flash II Digital Slide Scanner and analyzed with the Panoramic Viewer (3DHISTECH Ltd., Budapest, Hungary).

Immunolabeling for ATRX was considered as positive if tumor cells had nuclear labeling, whatever its intensity (1, 2 or 3), with no evidence of cytoplasmic labeling. Neoplasms were scored as negative for ATRX if there was no labeling. One tumor presenting cytoplasmic sequestration with a strong intensity was considered as interpretable. The internal controls (inflammatory and endothelial cells) had to be positive with a nuclear labeling; otherwise the case was considered as not interpretable.

Immunofluorescence

One hundred and twenty-seven tumors from the two cohorts were analyzed on tissue microarrays. Tissues were deparaffinized in xylene and rehydrated in a series of ethanol baths. For antigen retrieval, slides were incubated in DAKO Target Retrieval Solution, pH 9 (S236784-2, DAKO, Carpinteria, CA, USA), for 20 min in a microwave oven. The primary antibodies and dilutions (dilution in DAKO REAL antibody diluent, S202230-2, DAKO, Carpinteria, CA, USA) used to study ALT were as follows: anti-PML (1:200, PG-M3, RRID:AB_628162, sc-966, Santa-Cruz, Dallas, TX, USA) and anti-TERF2 antibody (1:200, HPA001907, RRID:AB_1080246, Sigma, St Louis, MO, USA). All primary antibodies were incubated for 1h at room temperature (RT). Secondary antibodies and dilutions used were as follows: anti-Mouse Immunoglobulins/FITC (1:400, F0479, Dako, Carpinteria, CA, USA) and anti-Rabbit IgG (H+L) Alexa Fluor® 594 conjugate (1:500, A-11072, RRID:AB_2534116, Thermo Fischer Scientific, Waltham, MA, USA). Slides were mounted with Vectashield/DAPI medium (H-1200-10, Vector Laboratories, Burlingame, CA, USA) and were then analyzed under a Nikon Eclipse 80i (Nikon, Melville, NY, USA) fluorescent microscope with appropriate filters. Pictures were captured using a Hamamatsu C4742-95 CCD camera (Hamamatsu, Hamamatsu City, Japan). For each sample, the 3 spots in the TMA were exhaustively screened for PML/TERF2 colocalization. A tumor was considered as ALT negative when no colocalized signal was observed in all screened nuclei. A tumor was considered as ALT positive when at least 10 colocalized signals were observed in the sample.

To study tryptase, tissue sections were blocked with 5% mouse serum PBS1X for 1h30 and incubated with mouse anti-tryptase antibody (1:300, ab2378, RRID:AB_303023, ABcam,

Cambridge, UK) for 1h at RT. Then Alexa Fluor Plus 594 goat anti-mouse secondary antibody (1:400, A-32742, RRID:AB_2762825, Thermo Fischer Scientific, Waltham, MA, USA) was incubated for 1h at RT. Slides were mounted using the Vectashield mounting medium plus DAPI (H-1200-10, Vector Laboratories, Burlingame, CA, USA). Images were acquired on a Zeiss Cell Observer microscope (Carl Zeiss, Oberkochen, Germany). Percentage of mast cells was assessed by counting the number of mast cells in 10 same size randomly localized regions of interest (ROI) in each tumor, divided by the total number of cells in these ROI determined by the number of nuclei count with Fiji (19).

ShRNA Knockdown of ATRX expression

shRNAs constructs targeting human or mouse *ATRX* were obtained from OriGene (Rockville, MD, USA). The 28 bp human sequence was 5'- CCTTCTAACTACCAGCAGTTGATATGAG -3' (TL306482A, OriGene, Rockville, MD, USA) and the 29 bp mouse sequence was 5'-CATCAAGTAGATGGTGTTCAGTTTATGTG -3' (TL502431B, OriGene, Rockville, MD, USA). A shRNA 29-mer scrambled shRNA was used as a negative control (TR30021V, OriGene, Rockville, MD, USA).

Production of lentiviruses

Lentiviruses were produced by co-transfection of pVSVg (RRID:Addgene_138479), psPAX2 (RRID:Addgene_12260) and shRNA construct in HEK293T cells. Co-transfection was performed by adding these plasmids, chloroquine at 0.025 mM (C6628, Sigma, St Louis, MO, USA), $CaCl_2$ at 0.125 M (C5050, Sigma, St Louis, MO, USA) and HeBS 1X (51558, Sigma, St Louis, MO, USA), HEK293T cells were then incubated at 37°C in a humidified chamber containing 5% CO_2. After 6 hours, HEK293T cell medium was changed with RPMI-1640 (524000-025, Life Technologies, Carlsbad, CA, USA) containing 10% of fetal bovine serum (S1810-500, Dutscher, Brumath, France).

Lentiviral transduction

HEK293T cell culture medium was filtered with a 0.45 µm PES filter and was mixed at 1:1 ratio with K7M2, MG63 or IB106 culture medium previously seeded. Polybrene (8 µg/ml, H9268, Sigma, St Louis, MO, USA) was also added with the virus. Infected cells were selected with puromycin and cells were sorted by FACS (BDFACSAria, BD Biosciences, San Jose, CA, USA) thanks to their GFP expression when vector with shRNA *ATRX* was integrated.

Western Blot analysis

Protein extracts were separated from each cell line with RIPA protein lysis buffer (R0278, Sigma, St Louis, MO, USA) containing 1X protease cocktail (P8340, Sigma, St Louis, MO, USA). Protein extracts were separated by electrophoresis on acrylamide gel (456-8085, Bio-Rad, Hercules, CA, USA) and transferred onto PVDF membrane. Then they were probed with antibodies against ATRX (1:1000, HPA001906, RRID:AB_1078249, Sigma, St Louis, MO, USA) or actin (1:5000, A5316, RRID:AB_476743, Sigma, St Louis, MO, USA). Proteins of interest were detected with HRP-conjugated horse anti-mouse IgG antibody (1:5000, 7076S, RRID:AB_330924, Cell Signaling Technology, Danvers, MA, USA) or HRP-conjugated goat anti-rabbit IgG antibody (1:5000, 7074S, RRID:AB_2099233, Cell Signaling Technology, Danvers, MA, USA) and visualized with the ECL prime Western blotting detection regent (RPN2236, GE Healthcare, Piscataway, NJ, USA), according to the manufacturer's recommendations and using the PXi system.

ALT specific c-circle detection

The C-circle assay, which partially detects single-stranded telomeric (CCCTAA)n DNA circles (C-circles) amplified by the Phi29 polymerase in the absence of dCTP, was performed as previously described [61].

Cell proliferation assay

Cells of each cell line with $ATRX^{KD}$ or $ATRX^{CT}$ were seeded onto a 96-well plate (3.10^3 cells/well). After 4 days, cell proliferation was evaluated by adding 20 µL of MTT (M2128, 5mg/mL, Sigma, St Louis, MO) to cell medium. Two hours later, medium was replaced by 100 µL of DMSO (5879, Sigma, St Louis, MO, USA) and the optic density (OD) of each well was read with a spectrophotometer at 570 and 630 nm. Live cell number was correlated to $\Delta OD = OD_{570nm} - OD_{630nm}$. Experiments were performed independently in triplicate three times.

Soft agar assay

Cells of each cell line with $ATRX^{KD}$ or $ATRX^{CT}$ were seeded (5000 cells/well) in 0.35 % agarose cell medium (16500-500, Invitrogen, Carlsbad, CA, USA) onto a 6-well plate containing a 0.5 % agar base. 0.5 mL of cell culture medium was added and changed every 3-4 days. After incubating for 3 to 4 weeks, colonies were visualized with 0.005 % crystal violet

staining (HT90132, Sigma, St Louis, MO, USA). Experiments were performed independently in triplicate four times.
